# Dietary L-glutamate, L-aspartate, and their combination supplementation modulated hematological, immune, and metabolic responses in weaned piglets challenged with enterotoxigenic *Escherichia coli*

**DOI:** 10.3389/fvets.2026.1938098

**Published:** 2026-10-20

**Authors:** Supatirada Wongchanla, Youngmin Park, Sangwoo Park, Shuhan Sun, Yanhong Liu

**Affiliations:** 1 Department of Animal Science, University of California, Davis, Davis, CA, United States; 2 Division of Animal Bioscience and Integrated Biotechnology, Gyeongsang National University, Jinju, Republic of Korea

**Keywords:** *Escherichia coli*, immunity, L-aspartate, L-glutamate, metabolomics, weaned piglet

## Abstract

L-glutamate (Glu) and L-aspartate (Asp) are functional amino acids involved in immune regulation and cellular metabolism. This study investigated their effects on hematological, immune, and metabolic responses in weaned pigs challenged with F18 enterotoxigenic *Escherichia coli* (ETEC). Weaned piglets (7.59 ± 1.11 kg BW) were randomly assigned to five treatments (*n* = 13): control (CON), 1% Glu (GLU), 1% Asp (ASP), 1% Glu and 1% Asp combination (GAC), and 50 mg/kg carbadox (ABX) supplementation. The experiment was conducted from d 0 to d 28. On d 21, GLU or ASP supplementation exhibited greater (*p* < 0.05) hemoglobin concentration, hematocrit, mean corpuscular volume, and mean corpuscular hemoglobin compared with CON-fed pigs. ASP and GLU supplementation also reduced (*p* < 0.05) red cell distribution width on d 28 compared with CON. Systemically on d 9, ASP or GAC supplementation reduced (*p* < 0.05) IL-8 concentrations compared with CON-fed pigs, while GLU or ASP supplementation lowered (*p* < 0.05) C-reactive protein compared with ABX-fed pigs. Compared with the other groups, haptoglobin concentration remained higher (*p* < 0.05) in CON-fed pigs on d 21 and 28. At the intestinal level, ASP or GLU supplementation increased (*p* < 0.05) jejunal *IL13* on d 28 compared with CON-fed pigs. In ileal mucosa, ASP supplementation reduced (*p* < 0.05) *IL8* expression on d 12, while GAC increased (*p* < 0.05) *IL4* and GLU increased (*p* < 0.05) *TGFB1* expression on d 28, compared with CON-fed pigs. Exploratory untargeted metabolomics identified treatment-associated differences (FDR < 0.2) in metabolites related to amino acid, carbohydrate, lipid, nucleotide, and redox metabolism. Pathway analysis identified enrichment (FDR < 0.2) of alanine, aspartate and glutamate metabolism, arginine biosynthesis, taurine and hypotaurine metabolism, citrate cycle, and glutathione metabolism. Overall, Glu, Asp, or their combination modulated hematological and inflammatory responses and were associated with metabolic changes during ETEC challenge. Combined supplementation did not consistently produce greater responses than individual supplementation. Glu and Asp produced favorable responses for several outcomes that were comparable to those observed in the antibiotic-treated group, supporting their potential as nutritional strategies during enteric challenge.

## 1 Introduction

The weaning transition in piglets induces psychological and physiological stress that can impair immune competence and disrupt metabolic homeostasis (1). This stress, combined with an underdeveloped gut immune system, increases susceptibility to bacterial infections, particularly enterotoxigenic *Escherichia coli* (ETEC) (2). Such infections further exacerbate weaning-associated challenges, contributing to post-weaning diarrhea (PWD) and compromised health. These effects are evident in clinical signs and in profound systemic and intestinal metabolic alterations (3).

ETEC colonizes the small intestine through fimbrial adhesins (e.g., F4 or F18) that enable attachment to enterocytes and subsequent toxin release (4). In addition to causing secretory diarrhea, ETEC infection triggers local and systemic immune responses characterized by elevated proinflammatory cytokine production and mucosal inflammation, further compromising intestinal barrier integrity (5). Concurrently, ETEC infection alters host metabolism, including increased energy demand, reduced nutrient utilization, and shifts in amino acid and lipid metabolism (6). Together, these immune and metabolic disturbances impair growth performance and resilience in affected pigs.

L-glutamate (Glu) and L-aspartate (Asp) are functional amino acids that play multifaceted roles in enterocyte metabolism and function (7). Both serve as intermediates in the citate cycle (also known as tricarboxylic acid cycle), supporting adenosine triphosphate (ATP) production. Glu is converted to α-ketoglutarate, an essential citate cycle intermediate that supports enterocyte energy generation and contributes to glutathione synthesis (8, 9). Asp provides oxaloacetate to the citrate cycle, fueling enterocyte metabolism, and supports nucleotide synthesis and cell proliferation (10). Additionally, both amino acids contribute to nitrogen metabolism and antioxidant defense.

Beyond metabolism, Glu and Asp modulate immune responses. Elevated luminal Glu concentrations have been reported to promote immune tolerance toward environmental antigens, potentially mitigating intestinal inflammation (11, 12). Asp has been shown to enhance immune function by fine-tuning inflammatory responses and reducing intestinal injury (13). Consistently, dietary supplementation with Glu or Asp in nursery pigs has been reported to alleviate intestinal inflammation, reduce oxidative stress, and improve resilience under pathogenic or oxidative challenges (14, 15).

In this study, we evaluated a nutritional strategy to mitigate PWD by supplementing weaned pig diets with Glu, Asp, or their combination. Given their roles in intestinal metabolism and immune regulation, we hypothesized that Glu or Asp would enhance host resilience during enteric challenge. Our previous pilot studies evaluated different inclusion levels of Glu and Asp and identified 1% supplementation as an effective inclusion level under ETEC challenge (16, 17). Building on these findings, the present larger-scale study used the selected 1% inclusion levels to further evaluate their effects and extend the investigation to combined Glu and Asp supplementation. While previous studies have demonstrated beneficial effects of Glu or Asp individually, the combined effects of Glu and Asp and their influence on systemic and intestinal immune-metabolic responses during different stages of ETEC infection remain poorly understood. Because Glu and Asp participate in interconnected metabolic pathways but may exert distinct physiological functions, their combined supplementation was investigated to determine whether it could provide additional or complementary effects beyond individual supplementation. The present study also evaluated responses during both the peak infection and recovery phases to characterize temporal physiological responses to supplementation. Therefore, the objective of this study was to evaluate and compare the effects of dietary Glu, Asp, and their combination on hematological indices, systemic and intestinal immune responses, and metabolic profiles in ETEC-challenged piglets.

## 2 Materials and methods

### 2.1 Animals, housing, experimental design, and diet

The protocol for this experiment was reviewed and approved by the Institutional Animal Care and Use Committee (IACUC #23529) of the University of California, Davis (UC Davis). A total of 65 weanling pigs (28 d old; 7.59 ± 1.11 kg body weight (BW)) were used in this experiment. The 6 sows and piglets used in this experiment were not given *E. coli* vaccinations, antibiotic injections, or antibiotics in creep feed. All piglets were genotyped for susceptibility to F18 ETEC infection based on the fucosyltransferase 1 (FUT1) gene polymorphism, using a polymerase chain reaction-restriction fragment length polymorphism (PCR-RFLP) assay. Briefly, genomic DNA was extracted from tail samples, and a specific region of the FUT1 gene (Forward primer, 5′-CTT AGA CCT GCT GGC CCT GT-3′ and Reverse primer, 5′-AGG ACA GGC AGC GTG ATG-3′) was amplified by PCR. The PCR product was then digested with a restriction enzyme, HinP1I, that targets the polymorphic site. Piglets displayed two distinct deoxyribonucleic acid (DNA) bands after digestion were identified as homozygous susceptible to F18 ETEC infection and were selected for this study. All piglets utilized in this experiment had not previously been infected with F18 ETEC. After weaning, pigs were housed in individual pens (0.61 m × 1.22 m) at the Cole facility at UC Davis. Pigs were contained in individual pens for 28 d, including a 7 d of adaptation period and 21 d following the first ETEC inoculation. All pigs were given free access to diet and water. Animal units were provided with ventilators and heaters to create the ideal thermoneutral zone for nursery piglets. Light was provided for 12 h a day from 07:30 to 19:30.

Piglets were randomly allocated to one of five treatments, with thirteen piglets per treatment (*n* = 13), using a randomized complete block design with weight and sex as blocks and the individual pig as the experimental unit. The treatments included: (1) basal diet with ETEC challenge (CON), (2) basal diet supplemented with 1% Glu with ETEC challenge (GLU), (3) basal diet supplemented with 1% Asp with ETEC challenge (ASP), (4) basal diet supplemented with 1% Glu and 1% Asp with ETEC challenge (GAC), and (5) basal diet supplemented with antibiotic growth promotor, carbadox at 50 mg/kg, with ETEC challenge (ABX). Glu and Asp were incorporated into the diets in free amino acid powder form and thoroughly mixed to ensure homogeneous distribution before feeding. Both Glu (Kosher/Orthodox Union, New York, NY, USA) and Asp (Anhui Sealong Biotechnology, Bengbu, Anhui, China) had a purity of ≥99%. The amino acid inclusion levels were selected based on our previous study (16).

All diets met the current estimates for nursery pig nutrient requirements (Supplementary Tables S1, S2), and the calculated standardized ileal digestible amino acid profiles are reported in Supplementary Table S3 (18). Diets did not include spray-dried plasma, antibiotics, or zinc oxide. Pigs began receiving their respective experimental diets on d 0 and remained on the assigned diets throughout the experimental period. All pigs were fed the experimental diets in a 2-phase feeding program, with weeks 1 and 2 serving as phase 1 and weeks 3 and 4 as phase 2. All pigs in the ETEC challenge treatments were orally inoculated with F18 ETEC for 3 consecutive days from d 7 before inoculation to d 9. The F18 ETEC was originally isolated from a field disease outbreak by the University of Montreal (isolate number: ECL22131). The F18 ETEC was given 10^10^ CFU per 3-mL dosage in phosphate-buffered saline (PBS), and it produces heat-labile toxin, and heat-stable toxin a and b. Our previous study (16) showed that this dose successfully caused diarrhea.

### 2.2 Sample collection procedures

The experiment included two euthanasia time points, on d 12 and d 28. Six pigs per treatment were euthanized on d 12 (*n* = 6), and the remaining pigs were euthanized on d 28 (*n* = 6–7). Pigs were anesthetized intramuscularly with a 1 mL mixture of 100 mg telazol, 50 mg ketamine, and 50 mg xylazine (2:1:1). Following anesthesia, each pig was euthanized with an intracardiac injection of 78 mg Fatal-Plus solution (sodium pentobarbital, MWI Animal Health, Visalia, CA, USA) per 1 kg of body weight. Approximately 10 cm of the intestine was dissected longitudinally and gently washed with PBS to remove luminal contents for intestinal mucosa collection on d 12 and d 28. Mucosa from the middle of the jejunum and ileum was scraped with a glass slide, promptly frozen in liquid nitrogen, and stored for gene expression and metabolomics analysis. Blood samples from all pigs were drawn from the jugular vein, with or without EDTA to collect whole blood and serum samples, respectively, on d 7 before inoculation, d 9, d 11, d 21, and d 28. Serum samples were stored at −80 °C until analysis of inflammatory biomarkers and metabolomics.

### 2.3 Assessment of complete blood count and serum inflammatory biomarkers

Total and differential blood cell counts were determined on whole blood samples by the Comparative Pathology Laboratory at UC Davis. The assay to optimally distinguish porcine blood was conducted using a multiparameter, automated programmed hematology analyzer (Drew/ERBA Scientific 950 FS Hematological Analyzer, Drew Scientific Inc., Miami, FL, USA). The concentrations of serum haptoglobin (Aviva Systems Biology Corp., San Diego, CA, USA), serum tumor necrosis factor-alpha (TNF-α), serum C-reactive protein (CRP), and interleukin 8 (IL-8) (R&D System Inc., Minneapolis, MN, USA) in serum samples were analyzed in duplicate by enzyme-linked immunosorbent assays (ELISA) according to the manufacturers’ instructions. The intensity of the color was measured at 450 nm with the correction wavelength set at 530 nm using a plate reader (BioTek Instruments, Inc., Winooski, VT, USA). The concentrations of each analyte in the tested samples were calculated based on a standard curve.

### 2.4 Quantitative analysis of intestinal immune-related gene expression

Reverse transcription quantitative polymerase chain reaction (RT-qPCR) was employed to assess gene expression in jejunal and ileal mucosa samples. Approximately 100 mg of each mucosa sample was homogenized with TRIzol reagent (Invitrogen; Thermo Fisher Scientific, Inc., Waltham, MA, USA). Subsequently, total ribonucleic acid (RNA) was extracted following the RNA extraction protocol recommendations provided by the reagent manufacturer. The High-Capacity Complementary Deoxyribonucleic acid (cDNA) Reverse Transcription Kit (Applied Biosystems; Thermo Fisher Scientific, Inc., Waltham, MA, USA) was used to generate cDNA from 1 μg of total RNA per sample in a 20 μL volume. To ensure RNA purity, absorbance ratios at 260 and 280 nm were measured with a Thermo Scientific NanoDrop 2000 Spectrophotometer (Thermo Scientific, Inc., Waltham, MA, USA). The mRNA expression levels of genes related to intestinal immune responses, mucosal immunity, and antioxidant defense were analyzed in intestinal mucosa. Immune-related genes included toll-like receptor 4 (*TLR4*), tumor necrosis factor-alpha (*TNFA*), interleukin-1 beta (*IL1B*), interleukin-6 (*IL6*), interleukin-8 (*IL8*), interferon-gamma (*IFNG*), interleukin-10 (*IL10*), interleukin-17A (*IL17A*), interleukin-22 (*IL22*), interleukin-4 (*IL4*), interleukin-13 (*IL13*), and transforming growth factor-beta 1 (*TGFB1*). Genes associated with mucosal immunity included polymeric immunoglobulin receptor (*PIGR*). Antioxidant- and redox-related genes analyzed in the ileal mucosa included glutathione peroxidase 4 (*GPX4*), glutathione reductase (*GSR*), glutamate–cysteine ligase catalytic subunit (*GCLC*), and solute carrier family 7 member 11 (*SLC7A11*). Data normalization was achieved using 18S ribosomal RNA (*18S rRNA*) and hypoxanthine phosphoribosyltransferase 1 (*HPRT1*) as housekeeping genes, which demonstrated stable expression across all samples and conditions, validating its suitability for normalization. Primers were designed according to published literature and commercially produced by Integrated DNA Technologies in Coralville, IA, USA. Each primer pair was verified and optimized through standard curve analysis, with amplification efficiencies ranging from 90 to 110%, ensuring reliability and consistency in RT-qPCR performance (Supplementary Table S4). A no template control (NTC) was included as a negative control to assess reagent contamination and the potential presence of genomic DNA. The analysis of relative gene quantification in comparison to the negative control was conducted using the 2^−ΔΔCT^ method. The RT-qPCR reaction conditions adhered to the protocols outlined in the published research (19).

### 2.5 Untargeted metabolomics analysis

Untargeted metabolomics analysis was conducted by the NIH West Coast Metabolomics Center at the University of California, Davis, using gas chromatography (Agilent 6,890 gas chromatograph controlled using Leco ChromaTOF software version 2.32, Agilent, Santa Clara, CA, USA) coupled with time-of-flight mass spectrometry (GC/TOF-MS) (Leco Pegasus IV time-of-flight mass spectrometer controlled using Leco ChromaTOF software version3 2.32, Leco, Joseph, MI, USA). Metabolite extraction was performed following procedures described previously (20). Briefly, frozen serum from d 11, jejunal mucosa, and ileal mucosa samples from d 12 (approximately 10 mg) were homogenized using a Retsch ball mill (Retsch, Newtown, PA, USA) for 30 s at 25 times/s. After homogenization, a prechilled (−20 °C) extraction solution (isopropanol/acetonitrile/water at the volume ratio 3:3:2, degassed with liquid nitrogen) was added at a volume of 1 mL/20 mg of sample. Samples were then vortexed and shaken for metabolite extraction. After centrifugation at 12,800 × g for 2 min, the supernatant was collected and divided into two equal aliquots and concentrated at room temperature for 4 h in a cold-trap vacuum concentrator (Labconco Centrivap, Kansas City, MO, USA). To separate complex lipids and waxes, the residue was re-suspended in 500 μL of 50% aqueous acetonitrile and centrifuged at 12,800 × g for 2 min. The resultant supernatant was collected and concentrated in the vacuum concentrator. Dried sample extracts were derivatized and mixed with internal retention index markers (fatty acid methyl esters with the chain length of C8 to C30). The samples were injected for GC/TOF analysis, and all samples were analyzed in a single batch. Data acquisition by mass spectrometry and mass calibration using FC43 (perfluorotributylamine) before starting analysis sequences. Metabolite identifications were performed based on the two parameters: (1) Retention index window ±2,000 U (around ±2 s retention time deviation), and (2) Mass spectral similarity plus additional confidence criteria as detailed below (Data processing). A detailed methodology for data acquisition and metabolite identification described in a previously published article by Fiehn et al. (20).

### 2.6 Metabolomics data processing and analysis

Metabolomics data were analyzed using MetaboAnalyst 6.0.^1^ Data were normalized using auto-scaling. Mass univariate analysis was performed using one-way ANOVA followed by Fisher’s least significant difference (LSD) test for pairwise comparisons, with adjusted *p* ≤ 0.05 considered significant. Multiple testing was controlled using the Benjamini–Hochberg false discovery rate (FDR) correction. Given the exploratory nature of the metabolomics analysis, an FDR < 0.2 was used as a predefined exploratory threshold to identify treatment-associated metabolic features for further biological interpretation (21, 22). Fold-change analysis was used to characterize the magnitude and direction of differences in individual metabolites among treatments. Metabolites with an FDR < 0.2 and a fold change >1.0 were considered upregulated and fold change >2.0 were considered strong upregulated, while those with a fold change <1.0 were considered downregulated. Partial least squares discriminant analysis (PLS-DA) was performed to characterize metabolic variation among treatment groups, and metabolites with a variable importance in projection (VIP) score >1 were considered for pathway analysis and metabolite set enrichment analysis. Pathways with a nominal *p* < 0.05, FDR < 0.2, and pathway impact >0.1 were identified as treatment-associated pathways for exploratory interpretation (23). Accordingly, metabolite and pathway findings meeting these criteria were interpreted as exploratory associations rather than definitive evidence of metabolic alterations.

### 2.7 Statistical analysis

Data normality was assessed using the UNIVARIATE procedure in SAS. All data, excluding metabolomics data, were analyzed using PROC MIXED of SAS (SAS Institute Inc., Cary, NC, USA). The statistical model included treatment as a fixed effect, blocks based on sex and body weight as random effects, and the individual pig as the experimental unit. All outcomes, except for metabolomics data, were predefined biological outcomes and were analyzed separately at each sampling time point. For hematological and inflammatory variables, sampling time points were also compared across all dietary treatments to evaluate changes over time following ETEC challenge. Treatment means were estimated using the LSMEANS statement, and pairwise treatment comparisons within each outcome and time point were performed using the PDIFF option with Tukey adjustment. Statistical significance and tendency were defined as *p* ≤ 0.05 and 0.05 < *p* ≤ 0.10, respectively.

## 3 Results

### 3.1 Hematological responses to dietary Glu, Asp, and their combination

ETEC infection increased (*p* < 0.05) white blood cell (WBC), neutrophil, lymphocyte, monocyte, eosinophil, and basophil counts, with values peaking (*p* < 0.05) on d 21 compared with d 7 (pre-inoculation; Supplementary Table S5). However, no differences in white blood cell profiles were observed across treatments within each time points.

ETEC infection increased (*p* < 0.05) red blood cell (RBC) count, mean corpuscular hemoglobin (MCH), mean corpuscular hemoglobin concentration (MCHC), platelet count and total protein concentration, but decreased (*p* < 0.05) hematocrit (HCT), mean corpuscular volume (MCV), red blood cell distribution width (RDW), and mean platelet volume (MPV) on d 9 and 11 compared with d 7 (Supplementary Table S5).

On d 9, pigs fed ASP tended to exhibit lower (*p* = 0.10) RDW compared with pigs fed GAC and ABX. On d 21, pigs fed GLU or ASP showed increased (*p* < 0.05) hemoglobin concentration, HCT, and MCH compared with pigs fed CON (Table 1). Pigs fed GLU, ASP, or GAC also had greater (*p* < 0.05) MCV than pigs fed CON, whereas pigs fed GLU or GAC exhibited increased (*p* < 0.05) RDW compared with CON on d 21. On d 28, RBC counts were lower (*p* < 0.05) in pigs fed ASP or GAC than in pigs fed ABX, while hemoglobin concentration and HCT were not affected. In addition, pigs fed ASP tended to have greater (*p* = 0.07) MCV compared with pigs fed CON and ABX, and pigs fed GLU or ASP had reduced (*p* < 0.05) RDW compared with pigs fed CON on d 28.

**Table 1:** Red blood cell profile of enterotoxigenic *Escherichia coli*-challenged weaned pigs.

Item^1^	CON	GLU	ASP	GAC	ABX	SEM	*p-*value
d 7							
RBC, 10^6^/μL	7.07	7.00	7.27	6.97	7.14	0.27	0.92
HGB, g/dL	7.95	7.88	8.15	7.65	7.47	0.32	0.50
HCT, %	31.02	29.40	32.13	32.37	30.89	1.45	0.49
MCV, fL	43.93	42.22	44.35	42.88	43.22	0.96	0.40
MCH, pg	11.32	11.01	11.42	10.99	10.43	0.47	0.60
MCHC, g/dL	25.11	26.31	25.72	25.33	24.08	0.73	0.27
RDW, %	29.59	30.15	28.23	30.28	31.19	0.92	0.24
Platelets, 10^3^/μL	442.39	531.55	406.01	425.29	394.55	54.24	0.15
MPV, fL	8.54	8.76	8.79	8.68	7.75	0.41	0.29
Total protein, g/dL	4.70	4.63	4.73	4.60	4.62	0.10	0.82
d 9							
RBC, 10^6^/μL	7.46	7.52	7.19	6.99	6.93	0.26	0.40
HGB, g/dL	8.14	8.54	8.33	7.84	7.70	0.38	0.49
HCT, %	31.20	30.94	30.87	29.03	29.01	1.13	0.44
MCV, fL	42.11	41.29	43.18	41.62	41.72	0.96	0.64
MCH, pg	10.99	11.42	12.32	11.25	11.03	0.43	0.20
MCHC, g/dL	26.04	27.62	27.11	26.89	26.31	0.62	0.32
RDW, %	28.09^ab^	28.78^ab^	26.48^b^	29.12^a^	29.52^a^	0.86	0.10
Platelets, 10^3^/μL	428.15	419.54	374.77	366.93	369.92	36.84	0.55
MPV, fL	8.47	8.01	8.62	8.16	7.81	0.34	0.33
Total protein, g/dL	5.04	4.86	4.81	5.14	4.96	0.15	0.56
d 11							
RBC, 10^6^/μL	7.89	8.00	7.33	7.39	7.11	0.35	0.33
HGB, g/dL	8.65	8.63	8.41	8.03	7.63	0.42	0.37
HCT, %	33.09	32.79	31.65	30.40	29.52	1.37	0.30
MCV, fL	42.11	41.22	43.66	40.20	41.53	1.07	0.19
MCH, pg	11.03	10.89	11.66	10.88	10.69	0.46	0.60
MCHC, g/dL	26.08	26.39	26.64	26.26	25.66	0.58	0.78
RDW, %	27.54	28.36	26.56	28.50	29.58	0.84	0.14
Platelets, 10^3^/μL	448.17	409.77	469.25	432.83	445.42	40.22	0.87
MPV, fL	8.56	7.91	8.86	8.49	7.79	0.42	0.14
Total protein, g/dL	5.34	5.14	4.75	5.41	5.16	0.24	0.13
d 21							
RBC, 10^6^/μL	6.94	7.61	6.82	6.64	6.26	0.37	0.18
HGB, g/dL	7.33^b^	9.30^a^	9.38^a^	7.82^ab^	7.27^b^	0.61	0.05
HCT, %	25.00^b^	32.97^a^	32.30^a^	27.47^ab^	24.98^a^	2.03	0.02
MCV, fL	36.00^c^	43.40^ab^	47.42^a^	41.32^b^	38.90^bc^	1.63	<0.01
MCH, pg	10.58^c^	12.31^ab^	13.76^a^	11.78^bc^	11.26^bc^	0.59	0.01
MCHC, g/dL	29.31	28.34	29.06	28.34	28.78	0.51	0.55
RDW, %	26.27^b^	33.22^a^	26.19^b^	31.96^a^	26.99^b^	1.73	0.02
Platelets, 10^3^/μL	491.72	346.73	415.05	368.38	388.38	46.90	0.23
MPV, fL	8.33	8.20	9.45	8.82	9.08	0.43	0.26
Total protein, g/dL	4.99	4.47	4.77	4.60	4.57	0.17	0.27
d 28							
RBC, 10^6^/μL	6.19^bc^	6.64^ab^	5.92^c^	6.15^c^	6.73^a^	0.17	0.01
HGB, g/dL	7.22	8.43	8.13	8.24	7.90	0.38	0.22
HCT, %	25.02	29.34	27.72	27.34	28.07	1.14	0.13
MCV, fL	40.47^b^	44.31^ab^	46.94^a^	44.42^ab^	41.71^b^	1.60	0.07
MCH, pg	11.67	12.74	13.80	12.70	11.77	0.58	0.11
MCHC, g/dL	28.73	28.71	29.35	28.53	28.03	0.63	0.70
RDW, %	32.17^a^	27.29^bc^	23.25^c^	30.08^ab^	28.57^ab^	1.56	0.01
Platelets, 10^3^/μL	388.21	324.71	328.38	342.71	378.04	45.59	0.72
MPV, fL	8.62	8.42	8.88	9.02	9.17	0.48	0.81
Total protein, g/dL	5.06	4.70	4.90	4.89	4.99	0.11	0.18

^1^RBC = red blood cell; HGB = hemoglobin; HCT = hematocrit; MCV = mean corpuscular volume; MCH = mean corpuscular hemoglobin; MCHC = mean corpuscular hemoglobin concentration; RDW = red cell distribution width; MPV = mean platelet volume. Each least squares mean represents 6 replicates (*n* = 6 pigs per treatment) from d 7 to d 12, and 6–7 replicates (*n* = 6–7 pigs per treatment) from d 12 to d 28. ^a,b,c^Means without a common superscript are different (*p* < 0.05).

### 3.2 Systemic inflammatory responses to dietary Glu, Asp, and their combination

ETEC infection increased (*p* < 0.05) serum TNF-α on d 11, 21, and 28, enhanced (*p* < 0.05) serum IL-8 on d 11. Additionally, ETEC infection also increased (*p* < 0.05) serum CRP from d 9 to 28 and serum haptoglobin on d 9 and 11, compared with d 7 (Supplementary Table S5). On d 7 before ETEC inoculation, pigs fed GLU or ASP showed greater (*p* < 0.05) serum IL-8 concentrations compared with CON and ABX (Table 2). CRP concentration also differed at d 7, with lower values (*p* < 0.05) observed in pigs fed GLU, ASP, GAC, or ABX compared with CON. No treatment effects were observed for serum TNF-α or haptoglobin on d 7.

**Table 2:** Serum inflammatory biomarkers of enterotoxigenic *Escherichia coli*-challenged weaned pigs.

Item^1^	CON	GLU	ASP	GAC	ABX	SEM	*p*-value
d 7							
TNF-α, pg./mL	4.49	6.22	4.41	8.02	5.78	1.26	0.17
IL-8, pg./mL	33.43^b^	127.46^a^	103.68^a^	70.57^ab^	11.95^b^	28.30	<0.01
CRP, μg/mL	11.05^a^	6.44^bc^	7.46^b^	5.11^bc^	3.33^c^	1.46	<0.01
Hp, μg/mL	1900.54	1144.97	1461.12	1323.11	1301.84	285.12	0.34
d 9							
TNF-α, pg./mL	6.71	6.09	6.47	10.84	5.48	1.78	0.23
IL-8, pg./mL	126.04^a^	74.48^ab^	35.35^bc^	11.93^c^	12.10^c^	21.55	<0.01
CRP, μg/mL	19.92^ab^	10.20^b^	14.13^b^	19.40^ab^	26.31^a^	4.18	0.04
Hp, μg/mL	2904.60	2248.15	1861.69	2035.61	2270.08	386.87	0.21
d 11							
TNF-α, pg./mL	8.44^b^	6.35^b^	8.57^b^	15.53^a^	6.59^b^	2.14	0.01
IL-8, pg./mL	154.97	134.95	218.83	111.66	141.84	44.71	0.50
CRP, μg/mL	19.07^a^	7.21^c^	11.63^bc^	8.78^bc^	14.64^ab^	3.56	0.01
Hp, μg/mL	3051.54	2968.25	1777.85	3356.62	2620.67	641.46	0.15
d 21							
TNF-α, pg./mL	13.55	10.78	12.84	20.87	6.63	3.72	0.14
IL-8, pg./mL	25.98	91.12	16.01	62.53	0.00	30.74	0.23
CRP, μg/mL	18.75	14.21	13.66	16.80	16.46	3.63	0.83
Hp, μg/mL	3710.65^a^	492.83^b^	207.47^b^	1649.04^b^	1436.74^b^	608.80	0.01
d 28							
TNF-α, pg./mL	12.13	19.41	17.40	11.45	8.28	4.96	0.23
IL-8, pg./mL	71.04	4.53	54.68	1.89	47.33	31.59	0.45
CRP, μg/mL	35.75	20.19	24.83	17.18	13.07	6.71	0.17
Hp, μg/mL	2973.85^a^	1049.89^b^	966.27^b^	964.48^b^	310.44^b^	268.90	<0.01

^1^TNF-α = tumor necrosis factor alpha; IL-8 = interleukin 8; CRP = C-reactive protein; Hp = haptoglobin. Each least squares mean represents 6 replicates (*n* = 6 pigs per treatment) from d 7 to d 12, and 6–7 replicates (*n* = 6–7 pigs per treatment) from d 12 to d 28. ^a,b,c^Means without a common superscript are different (*p* < 0.05).

After ETEC inoculation, pigs fed ASP, GAC, or ABX showed lower (*p* < 0.05) serum IL-8 compared with CON, while pigs fed GLU or ASP had reduced (*p* < 0.05) serum CRP than pigs fed ABX on d 9. On d 11, pigs fed GAC exhibited greatest (*p* < 0.05) serum TNF-α among all treatments. Pigs fed GLU, ASP, or GAC had lower (*p* < 0.05) serum CRP than CON and ABX. In addition, pigs supplemented with GLU, ASP, GAC, or ABX had reduced (*p* < 0.05) serum haptoglobin than pigs in CON on d 21 and 28. No differences were detected among treatments for TNF-α, IL-8, or CRP on d 21 and 28.

### 3.3 Intestinal immune responses to dietary Glu, Asp, and their combination

Results of gene expression in jejunal and ileal mucosa are present in Table 3. On d 12, pigs fed with GLU tended to have lower (*p* = 0.08) expression of jejunal *TLR4* than pigs fed GAC and ABX. Pigs fed GAC tended to show higher (*p* = 0.08) expression of jejunal *TNFA* compared with pigs fed with CON, ASP, and ABX. Also, GAC had the highest (*p* < 0.05) expression of jejunal *IL1B* compared with the other groups. No treatment effects were observed for *IL6, IL8, IL17A, IFNG, IL10*, or *PIGR* expression in jejunal mucosa on d 12. On d 28, pigs fed GLU, ASP, or GAC tended to express more (*p* = 0.06) jejunal *IL4* compared with pigs fed CON. In addition, jejunal *IL13* expression was increased (*p* < 0.05) in pigs fed GLU, ASP, or ABX compared with CON. No differences among treatments were observed for *IL17A, IL22, IL10, TGFB1*, or *PIGR* expression in jejunal mucosa on d 28.

**Table 3:** Relative abundance of mRNA expression of immune-related genes in jejunal and ileal mucosa of enterotoxigenic *Escherichia coli*-challenged weaned pigs.

Item^1^	CON	GLU	ASP	GAC	ABX	SEM	*p-*value
Jejunal mucosa, d 12							
*TLR4*	1.00^ab^	0.51^b^	0.82^ab^	1.23^a^	1.25^a^	0.23	0.08
*TNFA*	1.00^b^	1.18^ab^	0.96^b^	1.54^a^	0.88^b^	0.30	0.08
*IL6*	1.00	0.70	0.65	1.75	0.94	0.35	0.13
*IL1B*	1.00^b^	0.55^b^	0.54^b^	1.59^a^	0.82^b^	0.38	0.01
*IL8*	1.00	0.68	0.39	0.69	0.60	0.26	0.20
*IL17A*	1.00	0.59	0.55	0.96	0.80	0.22	0.27
*IFNG*	1.00	1.84	1.12	1.20	1.07	0.45	0.45
*IL10*	1.00	1.38	1.02	1.74	1.61	0.34	0.38
*PIGR*	1.00	0.75	0.76	0.83	0.50	0.22	0.47
Jejunal mucosa, d 28							
*IL17A*	1.00	1.01	0.91	0.84	1.16	0.24	0.83
*IL22*	1.00	0.50	0.99	0.60	0.68	0.26	0.29
*IL4*	1.00^b^	4.77^a^	4.15^a^	3.98^a^	3.58^ab^	0.65	0.06
*IL13*	1.00^c^	2.05^a^	1.67^ab^	1.21^bc^	1.65^ab^	0.27	<0.01
*IL10*	1.00	1.33	1.20	1.12	0.89	0.21	0.54
*TGFB1*	1.00	1.08	1.24	1.07	1.15	0.15	0.52
*PIGR*	1.00	1.44	1.22	1.45	0.87	0.31	0.39
Ileal mucosa, d 12							
*TLR4*	1.00	1.15	1.17	1.31	1.17	0.13	0.56
*TNFA*	1.00^ab^	0.86^bc^	1.03^a^	0.82^c^	0.92^abc^	0.09	0.08
*IL6*	1.00	0.98	1.01	1.08	0.84	0.16	0.80
*IL1B*	1.00^ab^	0.35^b^	0.39^b^	1.37^a^	0.41^b^	0.35	0.03
*IL8*	1.00^a^	0.95^ab^	0.50^b^	1.37^a^	1.09^a^	0.31	0.04
*IL17A*	1.00^b^	1.08^ab^	0.58^b^	1.66^a^	0.79^b^	0.22	0.03
*IFNG*	1.00	0.98	0.97	1.25	0.81	0.22	0.52
*IL10*	1.00	0.78	1.12	0.90	0.72	0.15	0.19
*PIGR*	1.00	1.00	0.74	1.13	0.86	0.30	0.58
*GPX4*	1.00	0.98	0.94	1.08	0.97	0.08	0.75
*GSR*	1.00	1.18	0.88	1.07	1.03	0.14	0.60
*GCLC*	1.00	1.24	0.98	1.17	1.11	0.17	0.69
*SLC7A11*	1.00^b^	1.17^ab^	0.69^b^	1.81^a^	1.20^ab^	0.25	0.06
Ileal mucosa, d 28							
*IL17A*	1.00	0.81	0.76	0.89	0.74	0.08	0.16
*IL22*	1.00	0.49	0.80	0.73	0.43	0.15	0.44
*IL4*	1.00^b^	0.70^b^	0.81^b^	1.71^a^	0.75^b^	0.18	0.01
*IL13*	1.00	0.95	1.32	0.85	1.53	0.56	0.39
*IL10*	1.00	0.61	0.61	0.65	0.86	0.12	0.13
*TGFB1*	1.00^bc^	1.41^a^	1.03^bc^	1.12^b^	0.94^c^	0.06	<0.01
*PIGR*	1.00	0.98	0.94	0.92	0.94	0.05	0.75
*GPX4*	1.00	1.27	1.20	1.20	1.38	0.12	0.41
*GSR*	1.00	1.00	1.02	1.06	1.07	0.11	0.97
*GCLC*	1.00	1.06	1.29	1.24	1.47	0.16	0.22
*SLC7A11*	1.00	0.94	1.06	1.08	1.25	0.20	0.77

^1^*TLR4* = Toll-like receptor 4; *TNFA* = tumor necrosis factor alpha; *IL6* = interleukin 6; *IL1B* = interleukin 1 beta; *IL8* = interleukin 8; *IL17A* = interleukin 17A; *IFNG* = interferon gamma; *IL10* = interleukin 10; *PIGR* = polymeric immunoglobulin receptor; *IL22* = interleukin 22; *IL4* = interleukin 4; *IL13* = interleukin 13; *TGFB1* = transforming growth factor beta 1; *GPX4* = glutathione peroxidase 4; *GSR* = glutathione reductase; *GCLC* = glutamate–cysteine ligase catalytic subunit; *SLC7A11* = solute carrier family 7 member 11. Each least squares mean represents 6 replicates (*n* = 6 pigs per treatment) on d 12, and 6–7 replicates (*n* = 6–7 pigs per treatment) on d 28. ^a,b,c^Means without a common superscript are different (*p* < 0.05).

On d 12, pigs fed GLU and GAC tended to express lower (*p* = 0.08) ileal *TNFA* than pigs in ASP, while pigs fed GAC tended to have lower (*p* = 0.08) ileal *TNFA* expression than pigs in CON. Pigs fed GAC had higher (*p* < 0.05) expression of ileal *IL1B* and *IL17* expression compared with pigs fed ASP and ABX on d 12. Additionally, pigs fed ASP showed lower (*p* < 0.05) ileal *IL8* expression than pigs fed CON, GAC, and ABX on d 12. Dietary treatment did not affect *TLR4, IL6, IFNG, IL10, PIGR, GPX4, GSR*, or *GCLC* expression in ileal mucosa on d 12. On d 28, pigs fed GAC exhibited the greatest (*p* < 0.05) ileal *IL4* expression among all treatments. The expression of ileal *TGFB1* was greater (*p* < 0.05) in pigs fed GLU than other treatments and the expression of *TGFB1* in ileal mucosa was lower (*p* < 0.05) in pigs fed ABX than GAC. No treatment effects were observed for *IL17A, IL22, IL13, IL10, PIGR, GPX4, GSR, GCLC*, or *SLC7A11* expression in ileal mucosa on d 28.

### 3.4 Metabolite alterations in response to dietary Glu, Asp, and their combination

A total of 414 metabolites (170 identified and 244 unidentified) were detected in jejunal mucosa on d 12. Aspartate abundance was higher and met the predefined exploratory criteria (VIP > 1, *p* < 0.05, FDR < 0.2) in pigs fed GLU or GAC compared with ABX-fed pigs. The PLS-DA score plot with 95% confidence intervals showed separation among the treatment groups, as illustrated in Supplementary Figures 1A,B. In ileal mucosa on d 12, 393 metabolites (161 identified and 232 unidentified) were detected, with greater glutaric acid abundance meeting the exploratory criteria (VIP > 1, *p* < 0.05, FDR < 0.2) in GAC-fed pigs compared with CON-fed pigs (Supplementary Figure 1C). In serum on d 11, 402 metabolites (142 identified and 260 unidentified) were detected, with no metabolites meeting the predefined exploratory criteria among treatments.

Fold changes of upregulated metabolites are presented in Table 4. Metabolites exhibiting fold changes > 2 were observed across serum d 11, jejunal mucosa d 12, and ileal mucosa d 12 in response to dietary treatments. In serum, GLU, ASP, and GAC supplementation consistently increased (*p* < 0.05, FDR < 0.2) monounsaturated fatty acids, including palmitoleic and oleic acids compared with pigs fed with CON. In addition, pigs fed GLU or GAC also increased taurine and linoleic acid compared with pigs fed with CON. In the jejunal mucosa, amino acid supplementation markedly elevated carbohydrate-related metabolites including maltose, lactose, and sucrose, as well as nucleotide-associated metabolites such as adenosine-5-monophosphate compared with pigs fed with CON. In the ileal mucosa, the largest fold changes were detected in disaccharides and related metabolites, with GLU-fed pigs showing increased sucrose and cellobiose abundance compared with pigs fed CON, ASP, GAC, and ABX. Pigs fed with GLU or ASP also increased dehydroascorbic acid compared with pigs fed with CON or GAC. In addition, GLU, ASP, or GAC supplementation increased glucose-6-phosphate, 2-monoolein, 5-aminovaleric acid compared with pigs fed ABX in ileal mucosa on d 12.

**Table 4:** Metabolite fold changes in serum, jejunal mucosa, and ileal mucosa of enterotoxigenic *Escherichia coli*-challenged weaned pigs.

Treatment comparison^1^	Serum, d 11		Jejunal mucosa, d 12		Ileal mucosa, d 12	
	Metabolite	Fold change^2^	Metabolite	Fold change^2^	Metabolite	Fold change^2^
GLU/CON	Palmitoleic acid	6.357	Maltose	5.487	Sucrose	10.621
	Taurine	5.548	Cis-10-heptadecenoic acid	2.926	Dehydroascorbic acid	7.632
	Oleic acid	4.202	Adenosine-5-monophosphate	2.328	Cis-10-heptadecenoic acid	3.819
	2-hydroxybutanoic acid	3.390			Beta-sitosterol	3.143
	N-acetylglycine	3.321			Cellobiose	2.945
	Linoleic acid	2.526			5-aminovaleric acid	2.654
	Phenylacetic acid	2.516			Pentobarbital	2.127
	Glycyl tyrosine	2.085			5-hydroxy-3-indoleacetic acid	2.104
					1-monoolein	2.099
ASP/CON	Palmitoleic acid	3.341	Gluconic acid lactone	11.605	Dehydroascorbic acid	5.287
	Oleic acid	2.530	Maltose	5.679	Inosine 5-monophosphate	2.511
	Phenylacetic acid	2.459	Lactose	2.534		
	Beta-sitosterol	2.125	Cellobiose	2.332		
	Indole-3-propionic acid	2.076	Panose	2.221		
GAC/CON	Beta-sitosterol	5.939	Lactose	7.921	Nicotinic acid	3.649
	Palmitoleic acid	5.729	Maltose	4.671	2-aminobutyric acid	2.913
	Taurine	3.697	Cadaverine	4.601	5-aminovaleric acid	2.653
	Oleic acid	3.632	Beta-sitosterol	3.547	Inosine 5-monophosphate	2.549
	Chenodeoxycholic acid	2.951	5-aminovaleric acid	3.439	Ribose	2.163
	5-aminovaleric acid	2.904	Panose	3.366	Cadaverine	2.146
	Linoleic acid	2.485	Nicotinic acid	3.175	1-monoolein	2.096
			Sucrose	3.121	Fructose-1-phosphate	2.043
			Stigmasterol	2.935		
			Chenodeoxycholic acid	2.777		
			Adenosine-5-monophosphate	2.730		
			Adenosine	2.520		
			Cholic acid	2.448		
			Putrescine	2.363		
			Daidzein	2.139		
			1-monostearin	2.106		
			UDP-N-acetylglucosamine	2.101		
			Pentobarbital	2.096		
			Alpha-aminoadipic acid	2.077		
			Homocystine	2.058		
ABX/CON	Palmitoleic acid	5.334	Lactose	6.154	Adenosine	6.358
	Cis-10-heptadecenoic acid	4.365	Cellobiose	4.901	1-monoolein	3.142
	Beta-sitosterol	3.595	Maltose	3.041	Dehydroascorbic acid	2.712
	Oleic acid	2.849	Gluconic acid lactone	2.082	Inosine 5-monophosphate	2.074
	Chenodeoxycholic acid	2.317	Cholic acid	2.038		
	Taurine	2.311	Malonic acid	2.009		
	N-acetylglycine	2.082				
GLU/ASP	Taurine	3.177	Adenosine	2.235	Sucrose	12.066
	N-acetylglycine	3.135	Cyanoalanine	2.209	Raffinose	11.907
	2-hydroxybutanoic acid	3.120	Proline	2.150	Cellobiose	9.550
			D-erythro-sphingosine	2.052	Cis-10-heptadecenoic acid	3.125
					Chenodeoxycholic acid	2.951
					Beta-sitosterol	2.777
					D-erythro-sphingosine	2.505
					Pentobarbital	2.296
					Pinitol	2.228
					5-methoxytryptamine	2.178
					Mucic acid	2.124
					Fructose	2.095
					2-monoolein	2.027
GLU/GAC	Malic acid	2.022	Histidine	3.638	Cellobiose	15.023
			Cis-10-heptadecenoic acid	2.900	Raffinose	11.167
			Glucose	2.653	Dehydroascorbic acid	10.931
			Asparagine	2.480	Sucrose	9.274
			Dehydroascorbic acid	2.458	Mucic acid	4.988
			Cyanoalanine	2.226	5-methoxytryptamine	4.649
					Saccharic acid	4.548
					Beta-sitosterol	3.823
					Galactinol	2.927
					Cis-10-heptadecenoic acid	2.895
					D-ribulose 5-phosphate	2.822
					Pinitol	2.488
					Ribonic acid	2.314
					Glycerol-alpha-phosphate	2.255
ASP/GAC	Cis-10-heptadecenoic acid	6.827	Mannose	35.550	Dehydroascorbic acid	7.573
	Pipecolinic acid	2.195	Gluconic acid lactone	7.561	Glycerol-alpha-phosphate	3.597
	Malic acid	2.007	Glucose	4.267	Saccharic acid	2.794
			Dehydroascorbic acid	3.611	Mucic acid	2.348
			5-methoxytryptamine	2.763	5-methoxytryptamine	2.135
			Taurine	2.168		
			Ribulose-5-phosphate	2.079		
			Sorbitol	2.078		
			Allantoic acid	2.007		
GLU/ABX	2-hydroxybutanoic acid	3.335	Cis-10-heptadecenoic acid	5.735	Sucrose	8.775
	Montanic acid	2.701	Guanine	2.175	Cellobiose	6.824
	Taurine	2.401			2-monoolein	5.391
	Lactose	2.096			5-aminovaleric acid	3.396
	Myo-inositol	2.061			Glucose-6-phosphate	3.352
	Cellobiose	2.054			5-methoxytryptamine	3.251
					D-ribulose 5-phosphate	3.209
					Dehydroascorbic acid	2.814
					Cis-10-heptadecenoic acid	2.638
					Chenodeoxycholic acid	2.516
					Ribonic acid	2.325
					Fructose-6-phosphate	2.251
					Beta-sitosterol	2.066
ASP/ABX	Indole-3-propionic acid	2.393	Gluconic acid lactone	5.573	Glucose-6-phosphate	3.375
			Taurine	2.947	2-monoolein	2.659
			Cis-10-heptadecenoic acid	2.871	5-aminovaleric acid	2.229
			Dehydroascorbic acid	2.705	Fructose-6-phosphate	2.180
			Aspartate	2.257	D-ribulose 5-phosphate	2.106
			3,6-anhydro-D-glucose	2.181		
GAC/ABX	Pentitol	2.609	Cadaverine	3.332	2-monoolein	3.669
	Lactose	2.140	Guanine	3.298	5-aminovaleric acid	3.393
	5-aminovaleric acid	2.105	Nicotinic acid	3.147	Glucose-6-phosphate	3.027
	Cellobiose	2.082	Alpha-aminoadipic acid	3.102	Nicotinic acid	2.669
			5-aminovaleric acid	2.780	2-aminobutyric acid	2.004
			Sucrose	2.759		
			Homocystine	2.465		
			Chenodeoxycholic acid	2.235		
			Stigmasterol	2.210		
			Putrescine	2.193		
			Aspartate	2.112		
			Beta-sitosterol	2.101		
			N-methyl-UMP	2.063		
			Pyrophosphate	2.018		

^1^CON = control; GLU = glutamate supplementation; ASP = aspartate supplementation; GAC = glutamate and aspartate combination supplementation; ABX; carbadox supplementation. Each treatment included 6 replicates. The treatment listed before the slash (/) represents the numerator, and the treatment after the slash represents the denominator.

^2^Fold change values greater than 2.0 indicate strong upregulation of metabolites between the treatment groups being compared.

### 3.5 Serum metabolic pathway alterations in response to dietary Glu, Asp, and their combination

Exploratory pathway enrichment analysis of serum on d 11 identified multiple treatment-associated pathways meeting the predefined criteria (*p* < 0.05, FDR < 0.2, impact > 0.1), as summarized in Table 5. When comparing CON vs. ASP, the pathways related to Arginine biosynthesis and Alanine, aspartate and glutamate metabolism were enriched, with additional enrichment observed in Galactose metabolism and Citrate cycle. When comparing CON vs. ABX, several carbohydrate- and energy-related pathways were enriched, including Starch and sucrose metabolism, Galactose metabolism, Citrate cycle, Alanine, aspartate and glutamate metabolism, and Glyoxylate and dicarboxylate metabolism. In the comparison of GLU vs. ASP, Taurine and hypotaurine metabolism, Alanine, aspartate and glutamate metabolism, and Arginine biosynthesis were enriched. When comparing GLU vs. GAC, Taurine and hypotaurine metabolism, Alanine, aspartate and glutamate metabolism, Phenylalanine metabolism, Histidine metabolism, Citrate cycle, and Arginine biosynthesis were enriched. Starch and sucrose metabolism, Citrate cycle, Galactose metabolism, and Glyoxylate and dicarboxylate metabolism were enriched when comparing GLU vs. ABX. When comparing ASP vs. GAC, Arginine biosynthesis, Citrate cycle, Starch and sucrose metabolism, Fructose and mannose metabolism, and Glyoxylate and dicarboxylate metabolism were enriched. In the comparison of ASP vs. ABX, multiple metabolic pathways were enriched, including Starch and sucrose metabolism, Alanine, aspartate and glutamate metabolism, Arginine biosynthesis, Citrate cycle, Glyoxylate and dicarboxylate metabolism, Galactose metabolism, and Glutathione metabolism. Finally, in the comparison of GAC vs. ABX, Galactose metabolism was enriched.

**Table 5:** Pathway enrichment analysis of serum in enterotoxigenic *Escherichia coli*-challenged weaned pigs on d 11.

Treatment comparison^1^	Impact^2^ > 0.1	*p* < 0.05	FDR^3^ < 0.2	Total Metab.^4^
Serum, d 11				
CON vs. ASP				
Arginine biosynthesis	0.426	<0.001	0.001	14
Alanine, aspartate and glutamate metabolism	0.423	<0.001	0.012	28
Galactose metabolism	0.181	0.003	0.070	27
Citrate cycle (TCA cycle)	0.165	0.009	0.183	20
CON vs. ABX				
Starch and sucrose metabolism	0.606	0.001	0.013	18
Galactose metabolism	0.226	<0.001	0.001	27
Citrate cycle (TCA cycle)	0.177	0.001	0.015	20
Alanine, aspartate and glutamate metabolism	0.164	0.004	0.047	28
Glyoxylate and dicarboxylate metabolism	0.110	0.001	0.013	32
GLU vs. ASP				
Taurine and hypotaurine metabolism	0.829	<0.001	0.009	8
Alanine, aspartate and glutamate metabolism	0.421	0.001	0.026	28
Arginine biosynthesis	0.362	<0.001	0.004	14
GLU vs. GAC				
Taurine and hypotaurine metabolism	0.829	0.007	0.081	8
Alanine, aspartate and glutamate metabolism	0.471	0.001	0.026	28
Phenylalanine metabolism	0.238	<0.001	0.018	10
Histidine metabolism	0.221	0.002	0.040	16
Citrate cycle (TCA cycle)	0.132	0.004	0.063	20
Arginine biosynthesis	0.122	<0.001	0.000	14
GLU vs. ABX				
Starch and sucrose metabolism	0.425	0.004	0.087	18
Citrate cycle (TCA cycle)	0.177	<0.001	0.035	20
Galactose metabolism	0.145	0.001	0.058	27
Glyoxylate and dicarboxylate metabolism	0.110	0.003	0.074	32
ASP vs. GAC				
Arginine biosynthesis	0.303	<0.001	0.010	14
Citrate cycle (TCA cycle)	0.209	0.001	0.016	20
Starch and sucrose metabolism	0.190	0.007	0.108	18
Fructose and mannose metabolism	0.131	0.009	0.122	20
Glyoxylate and dicarboxylate metabolism	0.107	0.001	0.015	32
ASP vs. ABX				
Starch and sucrose metabolism	0.560	0.001	0.009	18
Alanine, aspartate and glutamate metabolism	0.537	<0.001	0.008	28
Arginine biosynthesis	0.362	<0.001	0.000	14
Citrate cycle (TCA cycle)	0.209	0.001	0.011	20
Glyoxylate and dicarboxylate metabolism	0.190	<0.001	0.000	32
Galactose metabolism	0.155	0.003	0.032	27
Glutathione metabolism	0.115	0.004	0.033	28
GAC vs. ABX				
Galactose metabolism	0.184	0.000	0.000	27

^1^CON = control; GLU = glutamate supplementation; ASP = aspartate supplementation; GAC = glutamate and aspartate combination supplementation; ABX; carbadox supplementation. Each treatment included 6 replicates.

^2^Pathway impact value represents the cumulative contribution of matched metabolite nodes, as determined by pathway topology analysis.

^3^FDR = False Discovery Rate, a statistical method used to correct for multiple comparisons.

^4^Total Metab. = Total Metabolites, the total number of metabolites associated with a given pathway.

### 3.6 Jejunal mucosal metabolic pathway alterations in response to dietary Glu, Asp, and their combination

Exploratory pathway enrichment analysis of jejunal mucosa on d 12 revealed multiple enriched (*p* < 0.05, FDR < 0.2, impact > 0.1) metabolic pathways among dietary treatments as summarized in Table 6. When comparing CON vs. GLU, Galactose metabolism and Alanine, aspartate and glutamate metabolism were enriched. When comparing CON vs. GAC, Taurine and hypotaurine metabolism, Histidine metabolism, and Nicotinate and nicotinamide metabolism were most enriched. In the comparison of CON vs. ABX, Starch and sucrose metabolism and Galactose metabolism were enriched. When comparing GLU vs. ASP, Alanine, aspartate and glutamate metabolism, Glyoxylate and dicarboxylate metabolism, and Citrate cycle were enriched. In the comparison of GLU vs. GAC, Phenylalanine, tyrosine and tryptophan biosynthesis, Glycine, serine and threonine metabolism, and Alanine, aspartate and glutamate metabolism were most enriched. When comparing GLU vs. ABX, Starch and sucrose metabolism, Alanine, aspartate and glutamate metabolism, and Arginine biosynthesis were most enriched. In the comparison of ASP vs. GAC, Taurine and hypotaurine metabolism, Starch and sucrose metabolism, and Pentose phosphate pathway were most enriched. When comparing ASP vs. ABX, Taurine and hypotaurine metabolism, Alanine, aspartate and glutamate metabolism, and Arginine biosynthesis were most enriched. Finally, in the comparison of GAC vs. ABX, Starch and sucrose metabolism, Histidine metabolism, and Nicotinate and nicotinamide metabolism were most enriched.

**Table 6:** Pathway enrichment analysis of jejunal mucosa in enterotoxigenic *Escherichia coli*-challenged weaned pigs on d 12.

Treatment comparison^1^	Impact^2^ > 0.1	*P* < 0.05	FDR^3^ < 0.2	Total Metab.^4^
Jejunal mucosa, d 12				
CON vs. GLU				
Galactose metabolism	0.178	<0.001	0.019	27
Alanine, aspartate and glutamate metabolism	0.116	0.005	0.131	28
CON vs. GAC				
Taurine and hypotaurine metabolism	0.400	0.016	0.164	8
Histidine metabolism	0.221	0.007	0.093	16
Nicotinate and nicotinamide metabolism	0.194	0.006	0.093	15
Arginine biosynthesis	0.186	0.005	0.093	14
Galactose metabolism	0.184	<0.001	0.037	27
Glutathione metabolism	0.116	0.005	0.093	28
CON vs. ABX				
Starch and sucrose metabolism	0.544	<0.001	0.002	18
Galactose metabolism	0.184	0.003	0.105	27
GLU vs. ASP				
Alanine, aspartate and glutamate metabolism	0.340	<0.001	0.001	28
Glyoxylate and dicarboxylate metabolism	0.190	0.001	0.019	32
Citrate cycle (TCA cycle)	0.164	0.011	0.122	20
GLU vs. GAC				
Phenylalanine, tyrosine and tryptophan biosynthesis	1.000	0.008	0.076	4
Glycine, serine and threonine metabolism	0.536	0.033	0.177	34
Alanine, aspartate and glutamate metabolism	0.534	<0.001	0.010	28
Starch and sucrose metabolism	0.494	0.026	0.147	18
Arginine and proline metabolism	0.369	0.040	0.199	36
Glyoxylate and dicarboxylate metabolism	0.250	0.001	0.016	32
Histidine metabolism	0.221	0.019	0.115	16
Galactose metabolism	0.213	<0.001	0.010	27
Nicotinate and nicotinamide metabolism	0.194	0.016	0.115	15
Arginine biosynthesis	0.186	0.001	0.020	14
Glutathione metabolism	0.116	0.017	0.115	28
GLU vs. ABX				
Starch and sucrose metabolism	0.498	0.009	0.098	18
Alanine, aspartate and glutamate metabolism	0.421	0.004	0.065	28
Arginine biosynthesis	0.186	0.004	0.065	14
Fructose and mannose metabolism	0.161	0.001	0.030	20
Galactose metabolism	0.145	<0.001	0.001	27
ASP vs. GAC				
Taurine and hypotaurine metabolism	0.829	0.001	0.032	8
Starch and sucrose metabolism	0.471	0.010	0.131	18
Pentose phosphate pathway	0.169	0.002	0.062	23
Glyoxylate and dicarboxylate metabolism	0.143	0.008	0.128	32
Galactose metabolism	0.142	<0.001	0.032	27
Glutathione metabolism	0.116	0.005	0.098	28
ASP vs. ABX				
Taurine and hypotaurine metabolism	0.829	0.009	0.140	8
Alanine, aspartate and glutamate metabolism	0.421	0.001	0.049	28
Arginine biosynthesis	0.186	0.002	0.049	14
Glutathione metabolism	0.115	0.001	0.049	28
GAC vs. ABX				
Starch and sucrose metabolism	0.425	0.014	0.136	18
Histidine metabolism	0.221	0.010	0.111	16
Nicotinate and nicotinamide metabolism	0.194	0.008	0.108	15
Fructose and mannose metabolism	0.161	<0.001	0.007	20
Glutathione metabolism	0.123	0.008	0.108	28
Galactose metabolism	0.103	<0.001	0.006	27

^1^CON = control; GLU = glutamate supplementation; ASP = aspartate supplementation; GAC = glutamate and aspartate combination supplementation; ABX; carbadox supplementation. Each treatment included 6 replicates.

^2^Pathway impact value represents the cumulative contribution of matched metabolite nodes, as determined by pathway topology analysis.

^3^FDR = False Discovery Rate, a statistical method used to correct for multiple comparisons.

^4^Total Metab. = Total Metabolites, the total number of metabolites associated with a given pathway.

### 3.7 Ileal mucosal metabolic pathway alterations in response to dietary Glu, Asp, and their combination

Exploratory pathway enrichment analysis of ileal mucosa on d 12 revealed multiple enriched (*p* < 0.05, FDR < 0.2, impact > 0.1) metabolic pathways among dietary treatments as summarized in Table 7. When comparing CON vs. GLU, Phenylalanine, tyrosine and tryptophan biosynthesis, Taurine and hypotaurine metabolism, and Glycerolipid metabolism were most enriched. In the comparison of CON vs. ASP, Phenylalanine, tyrosine and tryptophan biosynthesis, Taurine and hypotaurine metabolism, and Phenylalanine metabolism were most enriched. In the comparison of CON vs. GAC, Phenylalanine, tyrosine and tryptophan biosynthesis, beta-Alanine metabolism, and Arginine biosynthesis were most enriched. In the comparison of CON vs. ABX, Phenylalanine, tyrosine and tryptophan biosynthesis, beta-Alanine metabolism, and Phenylalanine metabolism were most enrich. When comparing GLU vs. ASP, Alanine, aspartate and glutamate metabolism, Histidine metabolism, and Amino sugar and nucleotide sugar metabolism were most enriched. In the comparison of GLU vs. GAC, Taurine and hypotaurine metabolism, Pentose and glucuronate interconversions, and Ascorbate and aldarate metabolism were most enriched. In the comparison of GLU vs. ABX, beta-Alanine metabolism, Alanine, aspartate and glutamate metabolism, and Pentose phosphate pathway were most enriched. In the comparison of ASP vs. GAC, Alanine, aspartate and glutamate metabolism, beta-Alanine metabolism, and Arginine and proline metabolism were most enriched. When comparing ASP vs. ABX, Starch and sucrose metabolism, Alanine, aspartate and glutamate metabolism, and beta-Alanine metabolism were most enriched. Finally, in the comparison of GAC vs. ABX, Starch and sucrose metabolism, Amino sugar and nucleotide sugar metabolism, and Alanine, aspartate and glutamate metabolism were enriched.

**Table 7:** Pathway enrichment analysis of ileal mucosa in enterotoxigenic *Escherichia coli*-challenged weaned pigs on d 12.

Treatment comparison^1^	Impact^2^ > 0.1	*P* < 0.05	FDR^3^ < 0.2	Total Metab.^4^
Ileal mucosa, d 12				
CON vs. GLU				
Phenylalanine, tyrosine and tryptophan biosynthesis	1.000	0.004	0.082	4
Taurine and hypotaurine metabolism	0.829	0.017	0.172	8
Glycerolipid metabolism	0.330	0.008	0.120	16
Starch and sucrose metabolism	0.258	0.011	0.141	18
Pentose and glucuronate interconversions	0.169	0.012	0.141	19
Amino sugar and nucleotide sugar metabolism	0.121	0.004	0.082	42
CON vs. ASP				
Phenylalanine, tyrosine and tryptophan biosynthesis	1.000	0.003	0.072	4
Taurine and hypotaurine metabolism	0.429	0.012	0.137	8
Phenylalanine metabolism	0.357	0.019	0.187	10
Starch and sucrose metabolism	0.273	0.006	0.100	18
Histidine metabolism	0.221	0.004	0.088	16
Amino sugar and nucleotide sugar metabolism	0.138	0.002	0.072	42
CON vs. GAC				
Phenylalanine, tyrosine and tryptophan biosynthesis	1.000	0.004	0.118	4
Beta-Alanine metabolism	0.399	0.017	0.160	21
Arginine biosynthesis	0.303	0.005	0.118	14
Pyrimidine metabolism	0.191	0.017	0.160	38
CON vs. ABX				
Phenylalanine, tyrosine and tryptophan biosynthesis	1.000	0.004	0.105	4
Beta-Alanine metabolism	0.399	0.016	0.130	21
Phenylalanine metabolism	0.357	0.027	0.178	10
Fructose and mannose metabolism	0.239	0.014	0.130	20
Pentose phosphate pathway	0.213	0.003	0.103	23
Amino sugar and nucleotide sugar metabolism	0.196	0.023	0.164	42
Pentose and glucuronate interconversions	0.169	0.012	0.130	19
Starch and sucrose metabolism	0.153	0.011	0.130	18
GLU vs. ASP				
Alanine, aspartate and glutamate metabolism	0.534	0.011	0.115	28
Histidine metabolism	0.189	0.002	0.082	16
Amino sugar and nucleotide sugar metabolism	0.123	0.005	0.082	42
Arginine biosynthesis	0.122	0.001	0.082	14
Lysine degradation	0.112	0.014	0.123	30
GLU vs. GAC				
Taurine and hypotaurine metabolism	0.400	<0.001	0.036	8
Pentose and glucuronate interconversions	0.169	0.007	0.199	19
Ascorbate and aldarate metabolism	0.162	0.018	0.199	10
Pentose phosphate pathway	0.125	0.012	0.199	23
GLU vs. ABX				
Beta-Alanine metabolism	0.399	0.004	0.072	21
Alanine, aspartate and glutamate metabolism	0.224	0.008	0.110	28
Pentose phosphate pathway	0.213	<0.001	0.013	23
Pyrimidine metabolism	0.128	0.002	0.061	38
ASP vs. GAC				
Alanine, aspartate and glutamate metabolism	0.537	<0.001	0.001	28
Beta-Alanine metabolism	0.399	0.013	0.088	21
Arginine and proline metabolism	0.372	0.010	0.088	36
Pentose and glucuronate interconversions	0.241	0.010	0.088	19
Nicotinate and nicotinamide metabolism	0.194	0.005	0.088	15
Arginine biosynthesis	0.186	<0.001	0.001	14
Glyoxylate and dicarboxylate metabolism	0.140	0.007	0.088	32
Amino sugar and nucleotide sugar metabolism	0.134	0.017	0.100	42
Pyrimidine metabolism	0.128	0.012	0.088	38
Pentose phosphate pathway	0.125	0.017	0.100	23
ASP vs. ABX				
Starch and sucrose metabolism	0.628	<0.001	0.001	18
Alanine, aspartate and glutamate metabolism	0.534	0.014	0.124	28
Beta-Alanine metabolism	0.399	0.006	0.062	21
Pentose phosphate pathway	0.213	0.001	0.014	23
Pyrimidine metabolism	0.128	0.001	0.014	38
Arginine biosynthesis	0.122	0.002	0.030	14
GAC vs. ABX				
Starch and sucrose metabolism	0.292	<0.001	0.033	18
Amino sugar and nucleotide sugar metabolism	0.247	0.011	0.171	42
Alanine, aspartate and glutamate metabolism	0.226	0.002	0.067	28

^1^CON = control; GLU = glutamate supplementation; ASP = aspartate supplementation; GAC = glutamate and aspartate combination supplementation; ABX; carbadox supplementation. Each treatment included 6 replicates.

^2^Pathway impact value represents the cumulative contribution of matched metabolite nodes, as determined by pathway topology analysis.

^3^FDR = False Discovery Rate, a statistical method used to correct for multiple comparisons.

^4^Total Metab. = Total Metabolites, the total number of metabolites associated with a given pathway.

## 4 Discussion

Weaning represents a stressful transition that disrupt physiological homeostasis and impair immune competence in piglets. As a result, piglets become more susceptible to enteric pathogens such as ETEC. The combined challenge of weaning stress and pathogen exposure trigger inflammation, elevate oxidative stress, and drive metabolic adaptations to maintain systemic balance. In the present study, dietary supplementation with Glu, Asp, or their combination modulated hematological indices, as well as systemic and intestinal immune responses and metabolic profiles in nursery pigs challenged with ETEC.

### 4.1 Hematological responses modulated by Glu, Asp, or their combination supplementation

Hematological parameters are sensitive indicators of piglet health and feed efficiency, particularly under diarrheal and inflammatory conditions (24, 25). In the present study, supplementation with Glu or Asp influenced erythrocyte-related indices during the post-infection period. On d 21, pigs fed Glu or Asp exhibited increased hemoglobin, hematocrit, and mean corpuscular hemoglobin, indicating improved erythropoiesis and oxygen-carrying capacity during peak immune activity. The concurrent increase in mean corpuscular volume further suggested improved erythrocyte maturation or cell volume regulation, potentially lowering the risk of diarrhea-associated anemia (26). Red blood cell distribution width, a marker of erythrocyte size variability and anemia risk (27), also responded to dietary treatments. Reduced RDW observed in ASP-fed pigs at certain time points indicated a more uniform erythrocyte population and stabilized erythropoiesis during the post-challenge period. In contrast, a transient increase in RDW in pigs fed Glu and combination treatments on d 21 likely reflected adaptive erythrocyte turnover during immune stimulation. Importantly, Glu-fed pigs exhibited lower RDW on d 28, indicating improved erythrocyte uniformity during recovery and normalization of erythropoiesis as inflammation subsided. Glu and Asp may support erythropoiesis through their roles in intermediary metabolism, including nucleotide biosynthesis, energy production, and redox balance (8, 28). These metabolic functions are especially critical during infection, when nutrient demand and oxidative stress are elevated. These findings suggested that Glu or Asp supplementation may enhance hematological resilience and support oxygen transport under inflammatory stress, potentially reducing the risk of diarrhea-associated anemia in weaned piglets.

### 4.2 Systemic inflammatory responses modulated by Glu, Asp, or their combination supplementation

Beyond the immature immune system of weaned piglets, the combined stress and ETEC infection synergistically amplifies inflammation and immune activation, ultimately impairing health and growth performance (1). Although leukocyte responses were largely unaffected in the present study, ETEC challenge induced clear systemic inflammatory responses, as reflected by changes in circulating cytokines and acute-phase proteins. Importantly, dietary supplementation with Glu or Asp modulated these responses, indicating a role in supporting immune regulation during infection. Serum CRP, a sensitive marker of early inflammation (29), was reduced in amino acid–supplemented pigs both before and after ETEC challenge, and was even lower than in antibiotic-treated pigs on d 9. This reduction suggested improved control of the initial inflammatory response. Likewise, Asp and the combined amino acid treatment markedly decreased serum IL-8 concentrations during the peak diarrhea. Because IL-8 plays a key role in chemokine-mediated neutrophil recruitment during acute intestinal infection (30), its attenuation suggested that Asp may help temper excessive inflammatory signaling during the peak diarrhea. Interestingly, the GAC treatment subsequently increased serum TNF-α on d 11, indicating that the early reduction in IL-8 did not represent broad suppression of the inflammatory response. Rather, the contrasting temporal responses of IL-8 and TNF-α suggest that combined Glu and Asp supplementation differentially modulated inflammatory signaling across the progression of ETEC infection. Given the distinct biological functions and temporal regulation of these cytokines, further investigation is needed to determine the mechanisms underlying this response.

These beneficial responses became more pronounced during recovery (d 21 to 28). Haptoglobin remained elevated in CON-fed pigs through d 28, whereas pigs fed Glu, Asp, or their combination exhibited lower serum haptoglobin concentrations that were comparable to ABX-fed pigs. As a key acute-phase protein and indicator of disease severity (31), persistently elevated haptoglobin in control pigs suggested prolonged systemic inflammation, whereas the sustained reduction in amino acid-supplemented pigs suggests a more rapid resolution of the systemic acute-phase response during recovery. Although Glu and Asp did not consistently suppress early pro-inflammatory cytokines, their effects on haptoglobin indicate that their influence on systemic inflammation became more apparent during the later recovery period. Notably, responses to Glu and Asp supplementation were comparable to those observed with ABX for several systemic inflammatory outcomes. These findings support the potential of Glu and Asp as nutritional alternatives to in-feed antibiotics for selected physiological responses during ETEC challenge. However, further studies under different challenge and production conditions are warranted to determine whether these responses translate into reduced antibiotic use in practice.

### 4.3 Intestinal immune responses during peak infection modulated by Glu, Asp, or their combination supplementation

Dietary Glu and Asp also modulated local immune responses in the small intestine, highlighting their role in shaping mucosal immunity during ETEC challenge. At the peak of inflammation and diarrhea on d 12, Glu supplementation influenced innate immune signaling in a segment-specific manner. In the jejunum, Glu reduced *TLR4* expression, a key receptor involved in recognizing Gram-negative bacterial lipopolysaccharide (30). This reduction may reflect moderated pathogen recognition and a more controlled inflammatory response. In the ileum, Glu and the combined treatment reduced *TNFA* expression, suggesting attenuation of excessive pro-inflammatory signaling at the primary site of ETEC colonization (32). This finding aligns with previous reports that Glu supplementation suppresses the TLR4 signaling pathway in the jejunum of lipopolysaccharide-challenged weaned pigs (14). In contrast, pigs fed the combined Glu and Asp showed greater *IL1B, IL8*, and *IL17A* expression in the small intestine during peak diarrhea, indicating enhanced local immune activation (30, 33). Interestingly, the combined treatment increased *TNFA* expression in the jejunum, consistent with elevated systemic TNF-α on d 11, while reducing *TNFA* expression in the ileum on d 12. This segment-specific response indicates that combined supplementation differentially affected inflammatory gene expression between the jejunum and ileum. The biological mechanisms underlying these regional differences remain unclear and warrant further investigation. Such differential responses likely reflect regional variation in microbial exposure, immune cell distribution, and amino acid utilization along the small intestine. Further investigation into the interaction between free amino acid supplementation and the gut microbiome is warranted.

Asp supplementation exhibited a distinct immunomodulatory pattern. On d 12, ASP-fed pigs showed greater *TNFA* expression but reduced *IL8* expression in the ileal mucosa. Increased *TNFA* may indicate early macrophage activation, whereas reduced *IL8* suggests moderated neutrophil recruitment (30). These findings are consistent with previous studies reporting that Asp supplementation reduces intestinal pro-inflammatory cytokine expression and attenuates intestinal injury (34). This selective regulation implies a more balanced inflammatory response that supports pathogen clearance without promoting excessive chemokine-driven inflammation. Notably, the reduction in ileal *IL8* expression paralleled lower circulating IL-8 concentrations observed during the early post-infection phase, reinforcing the role of Asp in modulating both local and systemic chemokine signaling. These findings align with our previous observations (16), suggesting that Asp supplementation differentially modulated local and systemic inflammatory responses rather than uniformly suppressing inflammation. Moreover, Asp had been shown to promote immune cell activation and functional responsiveness (35).

Additionally, pigs fed the combined Glu and Asp treatment exhibited increased ileal SLC7A11 expression on d 12. As SLC7A11 regulates cystine uptake, glutathione synthesis, and redox balance (36), its increased expression may indicate changes in redox-related regulation during acute infection. This finding is consistent with previous studies showing that Glu or Asp supplementation alleviates oxidative stress-induced intestinal dysfunction in piglets (37). Collectively, these results suggest that Glu and Asp supplementation modulated genes associated with intestinal redox regulation during ETEC challenge.

### 4.4 Intestinal immune responses during recovery modulated by Glu, Asp, or their combination supplementation

During recovery phase, changes in the expression of genes associated with adaptive and regulatory immune responses were observed. In the jejunum, pigs fed Glu, Asp, or their combination exhibited increased expression of *IL4* and *IL13* compared with control. Similarly, in the ileum, the combined treatment enhanced *IL4* expression, while Glu supplementation increased *TGFB1* expression compared with control. IL-4 and IL-13 are Th2-associated cytokines that support tissue repair, mucosal healing, barrier restoration, and humoral immunity, whereas TGF-β plays a central role in immune regulation and mucosal tolerance (30). Previous studies have reported that elevated extracellular Glu may further promote Th2 polarization, enhancing humoral responses while limiting excessive inflammation (11, 38). Together, these cytokines function as key anti-inflammatory mediators that facilitate resolution of inflammation. The upregulation of these genes could suggest a transition from acute inflammatory responses toward immune resolution and tissue recovery in amino acid–supplemented pigs. Rather than broadly suppressing inflammation, Glu and Asp may appear to rebalance it, dampening early inflammatory signaling while supporting adaptive immunity during recovery. Consistent with the systemic findings, these results suggest that dietary Glu and Asp supplementation was associated with changes in both intestinal and systemic immune responses during ETEC challenge (12). By attenuating excessive early chemokine responses and enhancing Th2- and regulatory-associated pathways, these amino acids may enhance disease resilience, facilitate restoration of intestinal integrity, and act as effective immunomodulators in weaned piglets. However, these findings are based on mRNA expression and should be interpreted as transcriptional responses rather than direct evidence of functional immune activity. Because cytokine mRNA abundance does not necessarily correspond to protein concentration or bioactivity, protein-level measurements are needed to confirm the functional relevance of these responses.

Importantly, the combined supplementation did not consistently produce greater responses than individual Glu or Asp supplementation. Although GAC influenced several immune and metabolic outcomes, ASP alone produced favorable responses for several measured parameters, whereas GAC was associated with greater expression of some pro-inflammatory markers during the early phase of ETEC infection. For example, GAC increased serum TNF-α on d 11 and increased expression of several inflammatory-related genes during peak infection, despite reducing serum IL-8 earlier in the challenge. These findings suggest that combining Glu and Asp does not uniformly enhance the effects observed with either amino acid alone and may differentially influence the timing and magnitude of inflammatory responses. Therefore, the present findings do not provide clear evidence that combined supplementation is superior to individual supplementation. Further factorial and dose–response studies are needed to distinguish additive effects from responses related to the greater total supplemental amino acid supply in the combined treatment.

### 4.5 Exploratory metabolomic responses associated with Glu, Asp, or their combination supplementation

Stress and inflammation can profoundly reprogram metabolism in piglets, enabling adaptation to challenges such as ETEC infection (1, 17). In the present study, exploratory metabolomic analyses identified treatment-associated differences in systemic and intestinal metabolic pathways associated with amino acid metabolism, energy production, and redox homeostasis. However, Glu and Asp were provided as 99% pure crystalline amino acids by replacing corn in the experimental diets, resulting in differences in dietary amino acid and nitrogen supply among treatments. This limitation is particularly relevant to the combined treatment, which received a greater total amount of supplemental amino acids than the individual Glu or Asp treatments. Therefore, the observed metabolic responses cannot be attributed solely to the specific functional effects of Glu and Asp, as differences in nutrient and metabolic substrate supply may also have contributed to the observed metabolic profiles. Consequently, responses observed with the combined treatment may reflect the combined effects of Glu and Asp, the greater total supplemental amino acid supply, or both, which cannot be distinguished by the present experimental design. These considerations should be taken into account when interpreting the treatment-associated metabolic differences described below.

Across serum, jejunal mucosa, and ileal mucosa, Alanine, aspartate and glutamate metabolism was consistently enriched in amino acid-supplemented pigs. This pathway is central to nitrogen metabolism and serves as a key metabolic link between amino acid catabolism and the Citrate cycle (8, 39). Consistent enrichment of this pathway, together with the Citrate cycle and several carbohydrate-related pathways, including Galactose metabolism, Starch and sucrose metabolism, and Glyoxylate and dicarboxylate metabolism, indicates treatment-associated changes in pathways linking amino acid and carbohydrate metabolism during ETEC challenge. Because activation of immune and repair processes imposes substantial energetic demands on the host (40), these metabolic changes may be associated with processes supporting cellular function and tissue recovery under inflammatory conditions.

Specific metabolite changes further suggested treatment-associated alterations in metabolic profiles in amino acid-supplemented pigs. Increased jejunal aspartate and adenosine-5-monophosphate (AMP) concentrations in Glu- and combined-supplemented pigs, together with elevated ileal glutaric acid in pigs fed the combined treatment, were associated with pathways involved in amino acid catabolism, nucleotide metabolism, and mitochondrial energy metabolism (41). In addition, increased serum 2-hydroxybutanoic acid in Glu-fed pigs and elevated palmitoleic and oleic acids in Asp-fed pigs were consistent with alterations in metabolites related to substrate and energy metabolism during inflammatory stress (42, 43). Collectively, these exploratory findings demonstrate that Glu and Asp supplementation was associated with changes in metabolic pathways related to amino acid and energy metabolism during ETEC infection; however, these changes may reflect both their functional roles and their contribution as metabolic substrates.

Notably, Arginine biosynthesis was repeatedly enriched in serum and intestinal tissues of amino acid-treated pigs. Increased putrescine in pigs fed the combined treatment and elevated aspartate concentrations in Asp-supplemented pigs were consistent with the observed enrichment of this pathway. Arginine is a key precursor for nitric oxide synthesis and plays important roles in immune regulation, macrophage function, and vascular homeostasis (44, 45). Therefore, changes in arginine-related metabolism may be associated with the moderated inflammatory responses and hematological parameters observed in amino acid-supplemented pigs, particularly those receiving Asp.

Importantly, Glutathione metabolism and Taurine and hypotaurine metabolism were enriched in intestinal tissues, particularly in Asp- and combined amino acid-treated pigs. Glutathione is a major intracellular antioxidant that maintains redox balance during inflammation (46). Consistent with this, pigs fed the Glu and Asp combination exhibited increased metabolites associated with glutathione synthesis, including homocystine, nicotinic acid, and alpha-aminoadipic acid (47). Similarly, Asp supplementation increased taurine concentrations, supporting enrichment of Taurine and hypotaurine metabolism, which contributes to antioxidant defense, inflammatory regulation, and mitochondrial stability (48, 49). Additionally, increased dehydroascorbic acid in Glu- and Asp-supplemented pigs may reflect changes in antioxidant redox cycling during ETEC challenge (50). Changes in redox-related metabolism were also indicated by enrichment of the Pentose phosphate pathway and increased concentrations of glucose-6-phosphate, D-ribulose-5-phosphate, and fructose-6-phosphate, consistent with alterations in the pentose phosphate pathway, which contributes to NADPH generation for glutathione recycling and nucleotide synthesis (51).

Together, these exploratory findings suggest that dietary Glu and Asp supplementation was associated with differences in metabolites and pathways related to amino acid and energy metabolism, arginine biosynthesis, and redox homeostasis during ETEC challenge. These metabolic responses coincided with changes in hematological and inflammatory outcomes, suggesting potential interactions between nutrient supply, metabolism, and physiological responses during infection. However, the nutritional contribution of supplemental Glu and Asp, including differences in amino acid and nitrogen supply among treatments, should also be considered when interpreting these metabolic responses. In addition, pathway identification is sensitive to the selected FDR and pathway-impact thresholds. Therefore, pathways near these cutoffs should be interpreted cautiously and considered exploratory.

## 5 Conclusion

In conclusion, dietary Glu, Asp, or their combination modulated hematological, immune, and metabolic responses in ETEC-challenged weaned pigs. Glu and Asp supplementation was associated with favorable changes in erythrocyte-related parameters and inflammatory responses, while exploratory metabolomic analyses identified treatment-associated differences in metabolites and pathways related to amino acid and energy metabolism, arginine biosynthesis, and redox homeostasis. However, combined Glu and Asp supplementation did not consistently produce greater responses than individual supplementation, and the present findings do not demonstrate clear additive or synergistic effects. The nutritional contribution of supplemental Glu and Asp should also be considered when interpreting these responses. Collectively, these findings support further investigation of Glu and Asp as nutritional strategies for supporting physiological responses of weaned pigs during enteric challenge.

## Data Availability

The original contributions presented in the study are included in the article/Supplementary material, further inquiries can be directed to the corresponding author.

## Footnotes

1 https://www.metaboanalyst.ca.
