# Supplementary material for: Dietary L-glutamate, L-aspartate, and their combination supplementation modulated hematological, immune, and metabolic responses in weaned piglets challenged with enterotoxigenic Escherichia coli
Source: Front Vet Sci. 2026 Oct 20;13:1938098. doi: 10.3389/fvets.2026.1938098 (PMC13761760; doi:10.3389/fvets.2026.1938098)
Supplement: Supplementary file 1 [file Image_1.pdf]

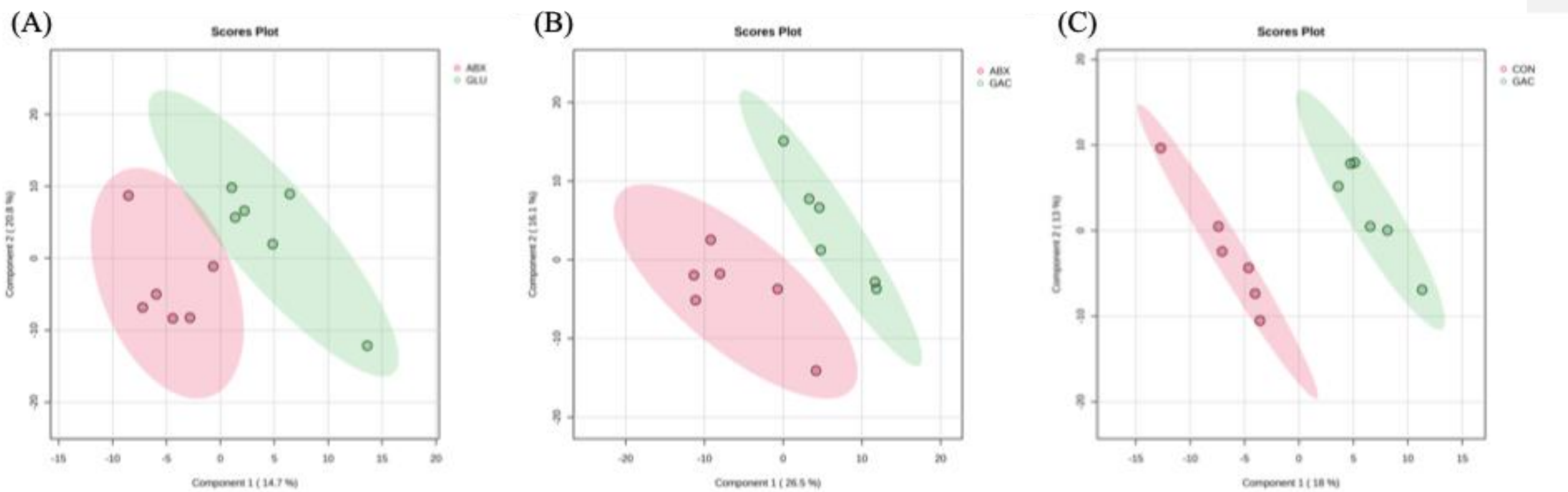

Supplementary Figure 1. 2D PLS-DA score plot of jejunal and ileal mucosa metabolites on d 12. Partial Least Squares Discriminant Analysis (PLS-DA) 2D score plot of the metabolites in jejunal and ileal mucosa samples from d 12 showed clear separation between the GLU and ABX groups in jejunal mucosa (A), GAC and ABX groups in jejunal mucosa (B), and CON and GAC groups in ileal mucosa (C). Each treatment included 6 replicates. CON = control; GLU = glutamate supplementation; GAC = glutamate and aspartate combination supplementation; ABX; carbadox supplementation.

Commented [YL1]: Suggest move this figure as supplementary
