# Supplementary material for: Dietary L-glutamate, L-aspartate, and their combination supplementation modulated hematological, immune, and metabolic responses in weaned piglets challenged with enterotoxigenic Escherichia coli
Source: Front Vet Sci. 2026 Oct 20;13:1938098. doi: 10.3389/fvets.2026.1938098 (PMC13761760; doi:10.3389/fvets.2026.1938098)
Supplement: Supplementary file 2 [file Table_1.docx]

**Supplementary Table 1.** Ingredient compositions of experimental diets, as-fed^1^

| **Ingredient, %** | **Control, Phase I** | **Control, Phase II** |
| --- | --- | --- |
| Corn | 52.03 | 53.89 |
| Dried whey | 15.00 | 10.00 |
| Soybean meal | 20.5 | 30.50 |
| Fish meal | 5.00 | - |
| Non-fat dried milk | 2.00 | - |
| Soy protein concentrate | 3.00 | - |
| Soybean oil | 2.00 | 2.00 |
| Limestone | 0.95 | 1.00 |
| Dicalcium phosphate | 0.25 | 0.90 |
| L-Lysine HCL | 0.35 | 0.39 |
| DL-Methionine | 0.13 | 0.12 |
| L-Threonine | 0.09 | 0.10 |
| Salt | 0.40 | 0.40 |
| Vit-mineral premix^2^ | 0.30 | 0.30 |
| TiO_2_ | - | 0.40 |
| Total | 100.00 | 100.00 |
| Analyzed nutrient, % as-is |  |  |
| Dry matter | 90.08 | 90.09 |
| Crude protein^3^ | 20.92 | 20.19 |
| Arg | 1.09 | 0.94 |
| His | 0.45 | 0.41 |
| Ile | 0.82 | 0.71 |
| Leu | 1.51 | 1.38 |
| Lys | 1.94 | 1.08 |
| Met | 0.36 | 0.31 |
| Phe | 0.82 | 0.76 |
| Thr | 0.72 | 0.63 |
| Trp | 0.23 | 0.23 |
| Val | 0.92 | 0.80 |
| Ala | 1.01 | 0.82 |
| Asp | 1.78 | 1.57 |
| Cys | 0.29 | 0.29 |
| Glu | 3.17 | 2.89 |
| Gly | 0.99 | 0.64 |
| Pro | 1.14 | 0.96 |
| Ser | 0.69 | 0.64 |
| Tyr | 0.55 | 0.52 |

^1^In each phase, four additional diets were formulated accordingly.

^2^Provided by the United Animal Health (Sheridan, IN, USA). Provided the following quantities of vitamins and micro minerals per kilogram of complete diet: Vitamin A as retinyl acetate, 11,136 IU; vitamin D3 as cholecalciferol, 2,208 IU; vitamin E as DL-alpha tocopheryl acetate, 66 IU; vitamin K as menadione dimethylprimidinol bisulfite, 1.42 mg; thiamin as thiamine mononitrate, 0.24 mg; riboflavin, 6.59 mg; pyridoxine as pyridoxine hydrochloride, 0.24 mg; vitamin B12, 0.03 mg; D-pantothenic acid as D-calcium pantothenate, 23.5 mg; niacin, 44.1 mg; folic acid, 1.59 mg; biotin, 0.44 mg; Cu, 20 mg as copper sulfate and copper chloride; Fe, 126 mg as ferrous sulfate; I, 1.26 mg as ethylenediamine dihydriodide; Mn, 60.2 mg as manganese sulfate; Se, 0.3 mg as sodium selenite and selenium yeast; and Zn, 125.1 mg as zinc sulfate.

^3^Calculated from total indispensable and dispensable amino acids.
