# Supplementary material for: Dietary L-glutamate, L-aspartate, and their combination supplementation modulated hematological, immune, and metabolic responses in weaned piglets challenged with enterotoxigenic Escherichia coli
Source: Front Vet Sci. 2026 Oct 20;13:1938098. doi: 10.3389/fvets.2026.1938098 (PMC13761760; doi:10.3389/fvets.2026.1938098)
Supplement: Supplementary file 3 [file Table_2.docx]

**Supplementary Table 2.** Analyzed amino acid concentrations (%, as-is) in Phase 2 experimental diets

| **Item**^1^ | **CON** | **GLU** | **ASP** | **GAC** | **ABX** |
| --- | --- | --- | --- | --- | --- |
| Arg | 0.94 | 1.09 | 1.09 | 1.11 | 1.10 |
| His | 0.41 | 0.46 | 0.45 | 0.47 | 0.47 |
| Ile | 0.71 | 0.81 | 0.78 | 0.82 | 0.82 |
| Leu | 1.38 | 1.52 | 1.51 | 1.52 | 1.55 |
| Lys | 1.33 | 1.30 | 1.01 | 1.34 | 1.24 |
| Met | 0.31 | 0.34 | 0.35 | 0.38 | 0.36 |
| Phe | 0.76 | 0.85 | 0.86 | 0.86 | 0.87 |
| Thr | 0.63 | 0.75 | 0.73 | 0.73 | 0.71 |
| Trp | 0.23 | 0.23 | 0.23 | 0.21 | 0.24 |
| Val | 0.80 | 0.90 | 0.84 | 0.91 | 0.91 |
| Ala | 0.82 | 0.92 | 0.89 | 0.90 | 0.90 |
| Asp | 1.57 | 1.86 | 2.73 | 2.84 | 1.85 |
| Cys | 0.29 | 0.34 | 0.28 | 0.31 | 0.33 |
| Glu | 2.89 | 4.24 | 3.21 | 5.05 | 3.33 |
| Gly | 0.64 | 0.73 | 0.70 | 0.73 | 0.73 |
| Pro | 0.96 | 1.04 | 1.09 | 1.04 | 1.06 |
| Ser | 0.64 | 0.73 | 0.78 | 0.72 | 0.73 |
| Tyr | 0.52 | 0.59 | 0.63 | 0.59 | 0.57 |

^1^CON = control diet; GLU = 1% Glu supplementation; ASP = 1% Asp supplementation; GAC = 1% Glu + 1% Asp supplementation; ABX = 50 mg/kg carbadox supplementation.
