# Supplementary material for: Dietary L-glutamate, L-aspartate, and their combination supplementation modulated hematological, immune, and metabolic responses in weaned piglets challenged with enterotoxigenic Escherichia coli
Source: Front Vet Sci. 2026 Oct 20;13:1938098. doi: 10.3389/fvets.2026.1938098 (PMC13761760; doi:10.3389/fvets.2026.1938098)
Supplement: Supplementary file 4 [file Table_3.docx]

**Supplementary Table 3.** Standardized ileal digestibility (%) of CP and AA of experimental diets fed in pigs on d 28^1^

| **Item**^2^ | | **CON** | **GLU** | **ASP** | **GAC** | **ABX** | ***SEM*** | ***P-*value**^3^ |
| --- | --- | --- | --- | --- | --- | --- | --- | --- |
| CP | | 77.03 | 78.24 | 81.69 | 83.09 | 83.17 | *2.33* | 0.257 |
| Indispensable AA | |  |  |  |  |  |  |  |
|  | Arg | 87.32 | 83.24 | 88.33 | 88.25 | 92.53 | *2.91* | 0.228 |
|  | His | 74.76 | 75.77 | 80.37 | 79.56 | 81.96 | *2.43* | 0.226 |
|  | Ile | 71.57 | 74.08 | 75.97 | 76.60 | 80.22 | *2.72* | 0.245 |
|  | Leu | 73.58 | 75.47 | 79.19 | 77.89 | 81.71 | *2.84* | 0.274 |
|  | Lys | 72.26^c^ | 75.67^bc^ | 73.80^c^ | 86.30^a^ | 79.77^b^ | *2.04* | **< 0.001** |
|  | Met | 81.60 | 83.93 | 86.32 | 86.61 | 88.20 | *2.17* | 0.248 |
|  | Phe | 71.45 | 73.96 | 77.53 | 76.01 | 80.27 | *2.73* | 0.197 |
|  | Thr | 72.70 | 75.66 | 79.04 | 76.88 | 81.25 | *2.83* | 0.261 |
|  | Trp | 87.15 | 86.74 | 89.68 | 86.89 | 92.10 | *1.90* | 0.229 |
|  | Val | 70.16 | 73.07 | 74.47 | 74.71 | 79.26 | *2.84* | 0.238 |
|  | Mean | 73.32 | 75.09 | 77.78 | 79.75 | 81.25 | *2.41* | 0.137 |
| Dispensable AA | |  |  |  |  |  |  |  |
|  | Ala | 69.89 | 73.47 | 74.06 | 73.18 | 77.00 | *3.30* | 0.578 |
|  | Asp | 67.25^d^ | 69.80^cd^ | 82.59^a^ | 81.65^ab^ | 75.59^bc^ | *2.15* | **< 0.001** |
|  | Cys | 59.38 | 63.20 | 59.77 | 62.80 | 69.75 | *3.20* | 0.222 |
|  | Glu | 70.36^b^ | 75.15^ab^ | 71.28^b^ | 81.05^a^ | 75.38^ab^ | *2.69* | **0.100** |
|  | Gly | 65.53 | 59.21 | 65.43 | 68.00 | 71.53 | *5.12* | 0.496 |
|  | Pro | 108.50 | 106.71 | 109.33 | 108.12 | 105.59 | *4.01* | 0.966 |
|  | Ser | 80.93 | 83.82 | 90.16 | 85.59 | 87.37 | *3.41* | 0.314 |
|  | Tyr | 74.53 | 76.21 | 79.96 | 78.65 | 80.92 | *2.29* | 0.233 |
|  | Mean | 75.62 | 77.22 | 80.70 | 82.64 | 80.86 | *2.46* | 0.294 |
| Total AA | | 74.65 | 76.29 | 79.50 | 81.45 | 81.24 | *2.38* | 0.218 |

^1^Each least squares mean represents 6-7 replicates (*n* = 6-7 pigs per treatment). Values for standardized ileal digestibility were calculated by correcting apparent ileal digestibility values for basal endogenous losses (g/kg of DMI), which were determined by feeding pigs an N-free diet (Almeida et al., 2013): CP, 22.40; Arg, 0.71; His, 0.28; Ile, 0.50; Leu, 0.85; Lys, 0.55; Met, 0.15; Phe, 0.53; Thr, 1.08; Trp, 0.22; Val, 0.71; Ala, 0.81; Asp, 1.22; Cys, 0.34; Glu, 1.38; Gly, 2.20; Pro, 4.61; Ser, 0.95; Tyr, 0.29.

^2^DM = dry matter; CP = crude protein; AA = amino acid; CON = control group; GLU = 1% Glu supplementation; ASP = 1% Asp supplementation; GAC = 1% Glu + 1% Asp supplementation; ABX = 50 mg/kg carbadox supplementation.

^3^Statistical significance and tendency were defined as *P* ≤ 0.05 and 0.05 < *P* ≤ 0.10, respectively.

^a,b,c,d^Means without a common superscript are different (*P* < 0.05).
