# Supplementary material for: Dietary L-glutamate, L-aspartate, and their combination supplementation modulated hematological, immune, and metabolic responses in weaned piglets challenged with enterotoxigenic Escherichia coli
Source: Front Vet Sci. 2026 Oct 20;13:1938098. doi: 10.3389/fvets.2026.1938098 (PMC13761760; doi:10.3389/fvets.2026.1938098)
Supplement: Supplementary file 5 [file Table_4.docx]

**Supplementary Table 4**. Sequences of oligonucleotide primers used for RT-qPCR assay

| Gene^1^ | Primer sequence (5’-3’) | Reference or Acc. No.^3^ |
| --- | --- | --- |
| *18s rRNA* | F: AGGAAAGCAGACATCGACCT | AB117609.1 |
|  | R: ACCTGGCTGTACTTCCCATC |  |
| *HPRT1* | F: GGACTTGAATCATGTTTGTG | NM_001032376.2 |
|  | R: CAGATGTTTCCAAACTCAAC |  |
| *GCLC* | F: AGTTCAACACGGTGGAGGACAATATG | (Gu et al., 2023) |
|  | R: CGGGCAGCCTAATCTGGGAAATG |  |
| *GPX4* | F: TCCTCATTGATAAGAACGGCTGTGTG | (Gu et al., 2023) |
|  | R: TAGCACGGCAGGTCCTTCTCTATG |  |
| *GSR* | F: GCCGACTGAACACCATCTATC | (Reddy et al., 2016) |
|  | R: GTGAGGAGCTGTGTACTTCTTC |  |
| *IL10* | F: TCGGCCCAGTGAAGAGTT TC | JQ687536.1 |
|  | R: GGAGTTCACGTGCTCCTTGA |  |
| *IL1B* | F: GGTGACAACAATAATGACCTGTTATTTG | NM_001302388.2 |
|  | R: GCTCCCATTTCTCAGAGAACCA |  |
| *IL13* | F: AAGTGGCCCAGTTCGTAAAAGA | (Li et al., 2019) |
|  | R: ACCCGTGGCGAAAAATCA |  |
| *IL17A* | F: CGGCTGGAGAAAGTGATGGT | NM_001005729.1 |
|  | R: CAGAAATGGGGCTGGGTCT |  |
| *IL22* | F: TTGACCAGTCCAACTTCCAGCAGC | XM_001926156.1 |
|  | R: GCAGCGCTCTCTCATATTGACTCC |  |
| *IL4* | F: TCACCTCCCAACTGATCCCA | NM_214123.1 |
|  | R: GCTCCATGCACGAGTTCTTT |  |
| *IL6* | F: GGGAAATGTCGAGGCTGTG | NM_214399 |
|  | R: AGGGGTGGTGGCTTTGTCT |  |
| *IL8* | F: TAGGACCAGAGCCAGGAAGA | (Li et al., 2019) |
|  | R: AGCAGGAAAACTGCCAAGAA |  |
| *INFG* | F: GAGCCAAATTGTCTCCTTCTAC | NM_213948.1 |
|  | R: CGAAGTCATTCAGTTTCCCAG |  |
| *PIGR* | F: GCCAAGGTCCTGGACAGATA | (Pasternak et al., 2015) |
|  | R: GTACACGGATTTCGGCTTCT |  |
| *SLC7A11* | F: TCTTTGTTGCCCTCTCCTGCTTTG | (Gu et al., 2023) |
|  | R: GAGTGTGTTTGCGGATGTGAATCATG |  |
| *TGFB1* | F: GAAGCGCATCGAGGCCATTC | NM_214015 |
|  | R: GGCTCCGGTTCGACACTTTC |  |
| *TLR4* | F: CATCCCCACATCAGTCAAGATACT | (Luo et al., 2015) |
|  | R: TCAATTGTCTGAATTTCACATCTGG |  |
| *TNFA* | F: CACCACGCTCTTCTGCCTACT | JF831365.1 |
|  | R: GACGGGCTTATCTGAGGTTTGA |  |

^1^*18S rRNA* = 18S ribosomal RNA; *HPRT1* = hypoxanthine phosphoribosyltransferase 1; *GCLC* = glutamate–cysteine ligase catalytic subunit; *GPX4* = glutathione peroxidase 4; *GSR* = glutathione reductase; *IL10* = interleukin 10; *IL1B* = interleukin 1 beta; *IL13* = interleukin 13; *IL17A* = interleukin 17A; *IL22* = interleukin 22; *IL4* = interleukin 4; *IL6* = interleukin 6; *IL8* = interleukin 8; *IFNG* = interferon gamma; *PIGR* = polymeric immunoglobulin receptor; *SLC7A11* = solute carrier family 7 member 11; *TGFB1* = transforming growth factor beta 1; *TLR4* = Toll-like receptor 4; *TNFA* = tumor necrosis factor alpha.

^2^F = forward; R = reverse.

^3^Acc. No. = accession number in GenBank database.

**References**

1. Gu, H., Liu, Y., Zhao, Y., Qu, H., Li, Y., Ahmed, A. A., Liu, H.-Y., Hu, P., & Cai, D. (2023). Hepatic Anti-Oxidative Genes CAT and GPX4 Are Epigenetically Modulated by RORγ/NRF2 in Alphacoronavirus-Exposed Piglets. *Antioxidants*, *12*(6), 1305. https://doi.org/10.3390/antiox12061305
2. Li, X., Mao, M., Zhang, Y., Yu, K., & Zhu, W. (2019). Succinate Modulates Intestinal Barrier Function and Inflammation Response in Pigs. *Biomolecules*, *9*(9), 486. https://doi.org/10.3390/biom9090486
3. Luo, Y., Van Nguyen, U., de la Fe Rodriguez, P. Y., Devriendt, B., & Cox, E. (2015). F4+ ETEC infection and oral immunization with F4 fimbriae elicits an IL-17-dominated immune response. *Veterinary Research*, *46*(1), 121. https://doi.org/10.1186/s13567-015-0264-2
4. Pasternak, J. A., Kent-Dennis, C., Van Kessel, A. G., & Wilson, H. L. (2015). Claudin‐4 Undergoes Age‐Dependent Change in Cellular Localization on Pig Jejunal Villous Epithelial Cells, Independent of Bacterial Colonization. *Mediators of Inflammation*, *2015*(1), 263629. https://doi.org/10.1155/2015/263629
5. Reddy, K. E., Lee, S. D., Kim, D. W., Oh, Y. K., Kim, M. J., Baek, C., Jeong, J., Kim, M. S., Kim, D. W., & Lee, H.-J. (2016). *Effects of Various Concentrations of Dietary Lead on Antioxidant Gene Expressions in Different Tissues of Pigs*. *3*(2).
