# Supplementary material for: Dietary L-glutamate, L-aspartate, and their combination supplementation modulated hematological, immune, and metabolic responses in weaned piglets challenged with enterotoxigenic Escherichia coli
Source: Front Vet Sci. 2026 Oct 20;13:1938098. doi: 10.3389/fvets.2026.1938098 (PMC13761760; doi:10.3389/fvets.2026.1938098)
Supplement: Supplementary file 6 [file Table_5.docx]

**Supplementary Table 5.** Total and differential white blood cell and red blood cell profiles of enterotoxigenic *Escherichia coli*-challenged weaned pigs

| **Item**^1^ | **d 7** | **d 9** | **d 11** | **d 21** | **d 28** | **SEM** | ***P*-value** |
| --- | --- | --- | --- | --- | --- | --- | --- |
| WBC, 10^3^/μL | 12.33^d^ | 15.16^bc^ | 15.71^b^ | 19.72^a^ | 13.38^cd^ | 1.13 | < 0.01 |
| Neu, 10^3^/μL | 5.45^c^ | 6.96^b^ | 7.18^b^ | 10.98^a^ | 8.23^b^ | 0.59 | < 0.01 |
| Lym, 10^3^/μL | 5.24^c^ | 5.36^bc^ | 5.99^ab^ | 6.77^a^ | 3.91^d^ | 0.50 | < 0.01 |
| Neu:Lym | 1.10^c^ | 1.28^c^ | 1.19^c^ | 1.69^b^ | 2.28^a^ | 0.10 | < 0.01 |
| Mono, 10^3^/μL | 0.73^b^ | 1.12^a^ | 1.09^a^ | 1.26^a^ | 0.88^b^ | 0.09 | < 0.01 |
| Eos, 10^3^/μL | 0.45^b^ | 1.33^a^ | 1.35^a^ | 0.40^b^ | 0.23^b^ | 0.10 | < 0.01 |
| Baso, 10^3^/μL | 0.03^d^ | 0.04^cd^ | 0.05^bc^ | 0.10^a^ | 0.07^b^ | 0.01 | < 0.01 |
| Neu, % | 45.02^c^ | 43.42^c^ | 44.44^c^ | 54.95^b^ | 60.82^a^ | 1.72 | < 0.01 |
| Lym, % | 43.82^a^ | 37.16^bc^ | 38.96^b^ | 34.75^c^ | 29.73^d^ | 1.53 | < 0.01 |
| Mono, % | 6.02^c^ | 8.48^a^ | 7.08^b^ | 6.83^bc^ | 6.97^bc^ | 0.64 | < 0.01 |
| Eos, % | 4.16^b^ | 9.87^a^ | 8.76^a^ | 2.44^bc^ | 1.67^c^ | 0.72 | < 0.01 |
| Baso, % | 0.22^b^ | 0.25^b^ | 0.31^b^ | 0.58^a^ | 0.50^a^ | 0.05 | < 0.01 |
| RBC, 10^6^/μL | 7.09^b^ | 7.22^ab^ | 7.54^a^ | 6.84^b^ | 6.31^c^ | 0.16 | < 0.01 |
| HGB, g/dL | 7.81 | 8.11 | 8.27 | 8.12 | 7.87 | 0.22 | 0.28 |
| HCT, % | 31.13^a^ | 30.21^ab^ | 31.48^a^ | 28.69^bc^ | 27.65^c^ | 0.76 | < 0.01 |
| MCV, fL | 43.32^a^ | 41.98^b^ | 41.78^b^ | 41.66^b^ | 43.83^a^ | 0.69 | < 0.01 |
| MCH, pg | 11.03^c^ | 11.39^bc^ | 11.04^c^ | 11.86^ab^ | 12.44^a^ | 0.27 | < 0.01 |
| MCHC, g/dL | 25.29^c^ | 26.80^b^ | 26.24^b^ | 28.32^a^ | 28.19^a^ | 0.37 | < 0.01 |
| RDW, % | 29.88^a^ | 28.38^b^ | 28.09^b^ | 29.24^ab^ | 28.57^ab^ | 0.53 | 0.03 |
| Platelets, 10^3^/μL | 433.90^ab^ | 392.88^bc^ | 440.56^a^ | 399.46^abc^ | 353.22^c^ | 23.83 | 0.02 |
| MPV, fL | 8.51^ab^ | 8.22^b^ | 8.31^b^ | 8.87^a^ | 8.90^a^ | 0.24 | 0.03 |
| Total protein, g/dL | 4.67^d^ | 4.98^ab^ | 5.14^a^ | 4.68^cd^ | 4.91^bc^ | 0.10 | < 0.01 |
| TNF-α, pg/mL | 5.94^c^ | 7.29^bc^ | 9.18^b^ | 13.59^a^ | 14.26^a^ | 1.39 | < 0.01 |
| IL-8, pg/mL | 68.36^b^ | 51.98^b^ | 152.78^a^ | 39.13^b^ | 35.63^b^ | 15.86 | < 0.01 |
| CRP, µg/mL | 6.93^d^ | 18.21^ab^ | 12.21^c^ | 15.87^bc^ | 21.75^a^ | 2.46 | < 0.01 |
| Hp, μg/mL | 1468.15^b^ | 2275.59^a^ | 2706.05^a^ | 1543.76^b^ | 1317.30^b^ | 299.85 | < 0.01 |

^1^WBC = white blood cell; Neu = neutrophil; Lym = lymphocyte; Mono = monocyte; Eos = eosinophil; Baso = basophil; Neu:Lym = neutrophil-to-lymphocyte ratio; RBC = red blood cell; HGB = hemoglobin; HCT = hematocrit; MCV = mean corpuscular volume; MCH = mean corpuscular hemoglobin; MCHC = mean corpuscular hemoglobin concentration; RDW = red cell distribution width; MPV = mean platelet volume; TNF-α = tumor necrosis factor alpha; IL-8 = interleukin 8; CRP = C-reactive protein; Hp = haptoglobin. Each least squares mean represents 65 replicates from d 7 to d 12, and 31 replicates from d 12 to d 28. ^a,b,c,d^Means without a common superscript are different (*P* < 0.05).
